# Multi-Property De Novo Drug Design Using Deep Learning-Based Knowledge Distillation and Reinforcement Learning

**DOI:** 10.3390/ijms27146125

**Published:** 2026-07-08

**Authors:** Liuying Wang, Zhao Lu, Lijuan Cui, Chang Liu, Yuting Qin, Shundan Feng, Dongxue Wang, Weixue Yin, Zheng Kang, Lei Cao

**Affiliations:** 1 School of Health Management, Harbin Medical University, Harbin 150086, China; wangliuying@hrbmu.edu.cn (L.W.); luzhao2019@163.com (Z.L.); 18379426915@163.com (L.C.); 15266291982@163.com (C.L.); seesunset1124@163.com (Y.Q.); 15085712511@163.com (S.F.); wdx18246119067@126.com (D.W.); yinwx0222@163.com (W.Y.); 2 Department of Biostatistics, School of Public Health, Harbin Medical University, Harbin 150086, China

**Keywords:** computer-aided drug design, de novo drug design, multi-property optimization, deep learning, reinforcement learning

## Abstract

Computer-aided de novo drug design has been widely explored for early-stage drug discovery, yet the multi-property optimization of novel molecules remains challenging. We aimed to develop a de novo drug design model to efficiently optimize multiple properties simultaneously. We developed a teacher–student-interaction deep learning model fine-tuned by reinforcement learning (TSItransRL) using bioactivity datasets (DRD2 and JNK3/GSK3β targets). A conditional transformer was pretrained as the teacher model to incorporate multi-property information. A vanilla transformer served as the student model and was subsequently optimized through interactive knowledge distillation and reinforcement learning. An evaluation was conducted using MOSES and conditional metrics on two tasks, specifically generating molecules with DRD2-targeting activity and generating molecules with dual JNK3/GSK3β-targeting activity, with the analyses including docking, the similarity ensemble approach (SEA), and scaffold novelty. TSItransRL achieved success rates of 98.36% and 98.90% for the DRD2 and JNK3/GSK3β tasks, respectively, with an internal diversity of 0.795, outperforming most baselines. The docking, SEA, scaffold, and ADMET analyses were used as exploratory in silico assessments to support the preliminary prioritization of selected generated molecules. TSItransRL provides an in silico framework for benchmark-level multi-property molecular generation and prioritization, combining interactive knowledge distillation with reinforcement learning to explore molecules that satisfy predefined predicted-activity, drug-likeness, and synthetic-accessibility criteria. The generated molecules should be regarded as computational candidates for a further medicinal-chemistry assessment, independent validation, and experimental testing rather than experimentally validated leads.

## 1. Introduction

Drug development is a complex, time-consuming, high-risk, and expensive process [1,2,3]. In clinical drug discovery, the goal of in silico de novo drug design is to discover novel molecules with specific desirable properties, such as bioactivity, drug-likeness, and synthetic accessibility. This process can be viewed as an optimization problem, seeking molecules that meet quantitative criteria [4,5]. Advancing de novo drug design may help improve the efficiency of early-stage lead identification by prioritizing candidate molecules with desired computational properties. Recently, various deep learning-based models, including transformer models, recurrent neural networks (RNNs), autoencoders, reinforcement learning (RL), and graph neural networks (GNNs), have been developed for this task, showing impressive results. However, multi-property optimization remains challenging due to factors like multiple property constraints [6], the need for efficient molecular representation learning [7,8], and the inefficient iterative modification of molecular structures in the vast chemical space [4]. In drug discovery, candidate molecules must simultaneously satisfy diverse criteria, such as potency, safety-related properties, and synthetic feasibility, before they can be considered for further development. Therefore, computational methods that jointly consider multiple properties may help prioritize molecules for subsequent experimental evaluation. With the rise of artificial intelligence (AI) in drug design, computer-aided drug design (CADD) technologies such as molecular docking, molecular dynamics simulations, and virtual screening have become widely studied tools for supporting early-stage molecular prioritization.

Current optimization techniques have shown a useful benchmark performance in de novo molecular generation and have produced computationally promising molecules. Nevertheless, the generation of molecules with multiple coexisting desirable properties better meets the requirements of drug discovery [9], as it reflects the need to balance the predicted activity, safety-related properties, and synthetic accessibility during early-stage molecular prioritization. To address this, recent models incorporate property constraints into structural representations for generative tasks. Examples include knowledge-distilled conditional transformers finetuned by reinforcement learning [10], conditional variational autoencoders (VAEs) imposing molecular properties in encoding and decoding [11], generative models composing molecules as substructure mixtures [12], and graph convolution policy networks using reward functions for constraints [13]. Effective molecular representation learning is also essential [9], with methods converting molecules to multidimensional continuous representations for chemical space exploration. Notable studies include a variational autoencoder (VAE) trained on properties [4], geometry-enhanced representations utilizing the topology and geometry information of molecules [8], a graph VAE for molecular graphs [14], and hierarchical graph modeling with contrastive learning based on two-level graph similarities [15]. Additionally, efficient exploration strategies are crucial [16], employing reinforcement learning [17], evolutionary explorers [16], and an evolution search framework [18] to navigate chemical space. Despite these advances, existing conditional generators often require users to specify feasible property ranges [9,11,19,20,21,22,23], potentially generating invalid molecules for unrealistic values. Moreover, molecular representation learning directly impacts the generation performance, warranting more efficient strategies [7,19,24,25,26,27,28]. Exploration methods, while effective, could be optimized for faster convergence to optimal regions; for instance, reinforcement learning requires substantial steps with predefined rewards [10], and genetic algorithms demand manual mutation and crossover rules [4,29,30]. These gaps limit the reliability and efficiency of computational molecular generation, especially when diverse molecules satisfying multiple predefined criteria are required.

In this study, we propose a teacher–student interaction transformer fine-tuned by reinforcement learning (TSItransRL) for multi-property de novo molecular generation. The novelty of TSItransRL lies in the staged integration of conditional generation, interactive knowledge distillation, and reward-driven refinement, rather than in the isolated use of any single component. Specifically, TSItransRL adopts an asymmetric teacher–student design: a conditional transformer teacher is pretrained on target-specific labeled datasets to learn multi-property constraints, whereas a vanilla transformer student is pretrained on PubChem to capture a broad chemical-space prior. During interactive knowledge distillation, the teacher generates positive multi-property pseudo-samples to guide the student, while the student generates molecules without explicit condition labels and feeds newly evaluated labeled molecules back to update the teacher. This bidirectional interaction differs from conventional one-way knowledge distillation and enables the student model to progressively acquire a target-specific multi-property generation ability without requiring conditional inputs during inference. Finally, the distilled student model is further refined by proximal policy optimization (PPO)-based reinforcement learning with Kullback–Leibler (KL) regularization, which biases generation toward high-reward molecules while limiting excessive deviation from the learned molecular distribution. TSItransRL was evaluated using a primary dopamine type 2 receptor (DRD2)-targeting benchmark and a supplementary dual c-Jun N-terminal kinase 3/glycogen synthase kinase 3 beta (JNK3/GSK3β)-targeting benchmark. The results support its benchmark-level performance for in silico multi-property molecular generation, while further independent and experimental validation is required to establish the practical drug-discovery utility.

## 2. Results

### 2.1. TSItransRL Model Framework

**Figure 1:**
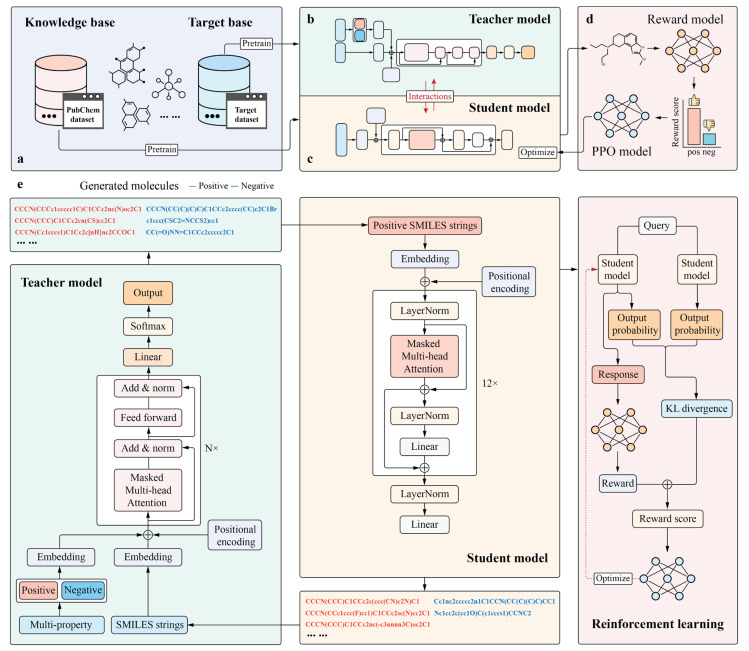
The workflow of TSItransRL. (**a**) The knowledge and target database for the pre-training of the student model and teacher model. (**b**,**c**) The teacher–student interaction knowledge distillation. (**d**) The fine-tuning of the student model through reinforcement learning. (**e**) The architecture of the teacher model and the student model, and the workflow of the reinforcement learning fine-tuning.

The workflow for TSItransRL is illustrated in Figure 1. The framework consists of three essential components: the teacher model, the student model, and the reinforcement learning model. First, the teacher model (Figure 1b) was pretrained on target-specific molecular datasets using paired samples composed of a simplified molecular-input line-entry system (SMILES) sequence and a binary condition label, denoted as (SMILES, c). The label c indicates whether a molecule satisfies all predefined task-specific property criteria, including the target activity, quantitative estimate of drug-likeness (QED), and synthetic accessibility (SA), as defined in Section 4.2. Meanwhile, the student model (Figure 1c) was pretrained on SMILES strings from the PubChem dataset (knowledge base, Figure 1a), without using condition labels, to learn the syntax and general distribution of valid chemical compounds. During interactive knowledge distillation, the teacher model was conditioned on the positive label (c = 1) to generate candidate molecules. These molecules were evaluated using the same predefined criteria, and only valid molecules satisfying all criteria were retained as positive pseudo-samples to fine-tune the student model. Subsequently, the student model generated molecules using only the start token <s> or partial SMILES tokens as input, without explicit condition labels. The student-generated molecules were evaluated and assigned binary labels according to the same criteria, and the resulting labeled samples were used to update the teacher model. This interaction continued until the student model achieved a stable performance in generating molecules with desired properties (see details in Section 4). Finally, the distilled student model was fine-tuned by reinforcement learning (RL) (Figure 1d) and used for molecular generation.

In practice, molecules with multiple desirable constraints (positive molecules) account for only a small proportion of all the related molecules (Table 1). Existing data-driven generative models often require training on numerous molecules with multiple desirable properties to learn effective representations. To mitigate this problem, the teacher model in TSItransRL generates large amounts of novel positive molecules as a new knowledge base, enabling the student model to capture rich information about positive molecules gradually through the teacher–student interaction process. Additionally, Kullback–Leibler (KL) divergence was introduced as part of the reward function to ensure that the distribution of the RL-adjusted student model remains similar to the pre-finetuned student model. The generated molecules were evaluated by a multiple-property reward function, with comprehensive reward scores used to update the student model via proximal policy optimization (PPO). This strategy prevents distribution collapse into few regions in chemical space and yields enhanced novelty and diversity.

### 2.2. The Advantages of Teacher–Student Model Interaction

Due to the superior performance of transformers in natural language learning, they were adopted as both the teacher and student models. Previous studies have demonstrated the superior performance of conditional transformers in utilizing structure–property relationships for molecular generation. Therefore, a modified conditional transformer was used as the teacher model, in which multiple properties were converted into a binary constraint and represented as a conditional token, while a vanilla transformer was used as the student model. To evaluate whether knowledge interaction improved the benchmark-level generation performance, we conducted the initial teacher–student model interaction (init-TS) and compared it with a conditional transformer (c-transformer), distilled likelihood (DL), distilled molecules (DM), initial-teacher model (init-T) and initial-student model (init-S) (see details in Section 4).

The findings of the DRD2-targeting task (task 1) are illustrated as the primary example. After training, a total of 5000 molecules were generated by each model and evaluated using MOSES [10] metrics (Table 2). While all models in the first four rows could yield positive molecules using conditional tokens, positive molecules only accounted for a small proportion (17~25.4%) of the generated molecules. Notably, the success rate of the student model (init-TS) increased to 89.5% when the knowledge interaction between init-T and init-S was conducted, indicating that the knowledge interaction effectively biases the probability distribution toward positive molecules.

**Figure 2:**
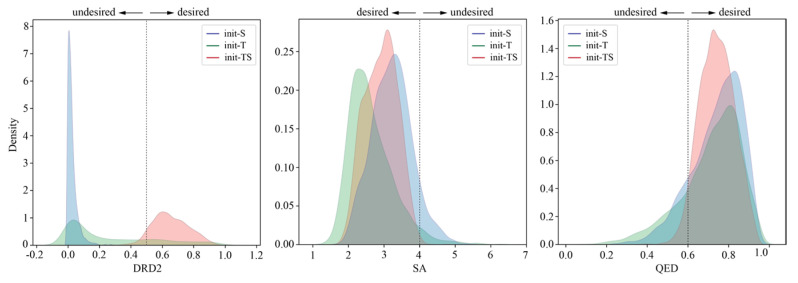
The advantage of teacher–student interactive knowledge distillation. Distributions of the molecular properties for the molecules generated by init-S, init-T and init-TS.

Compared with the evaluated baseline methods (c-transformer, DL and DM), the init-TS achieved higher validity and success rates under the benchmark criteria (Table 2). The knowledge interaction allows the teacher–student model to process numerous novel positive molecules, enabling the student model to capture rich information about positive molecules. The generated molecular distributions for three property constraints (Figure 2) indicate that init-TS effectively generates desired molecules. The supplementary dual JNK3/GSK3β-targeting benchmark (task 2) showed a similar trend in the teacher–student model interaction, as detailed in Supplementary Figure S1 and Table S1.

### 2.3. The Teacher–Student Model Interaction Analysis

**Figure 3:**
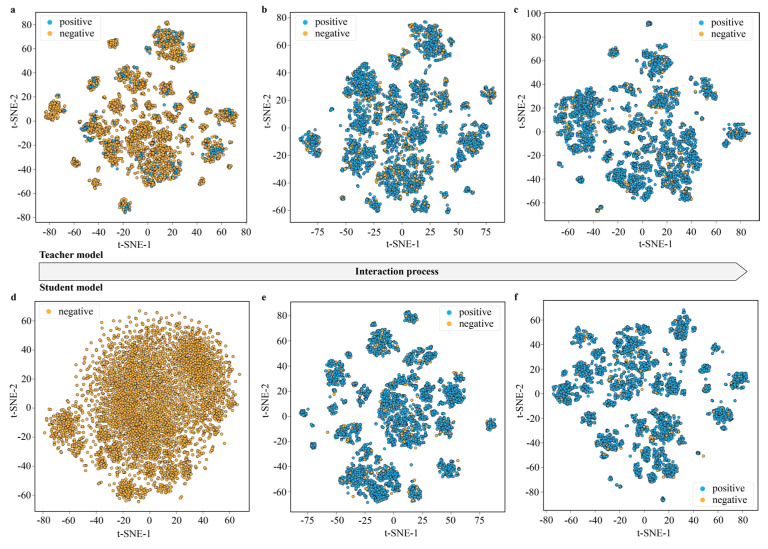
Visualization of molecular representation space during knowledge interaction. (**a**–**f**) The molecular representation for the molecules generated by the teacher model and student model, respectively, at the initial, 4th and 7th rounds of interaction.

To explore how interaction rounds affect model performance, we conducted a sensitivity analysis over seven rounds of knowledge interaction, with a three-fold evaluation after each round. As shown in Table 3, both the teacher and student models achieved an improved performance as the number of interaction rounds increased, showing significant improvements in validity, SNN, and success rates while maintaining other promising metrics. The visualization of the chemical representation space (Figure 3) not only demonstrates increased proportions of positive molecules, but also indicates that the interaction process avoids local optima. The JNK3/GSK3β-targeting task results (Supplementary Table S2 and Figure S2) showed a similar trend in the second benchmark.

**Figure 4:**
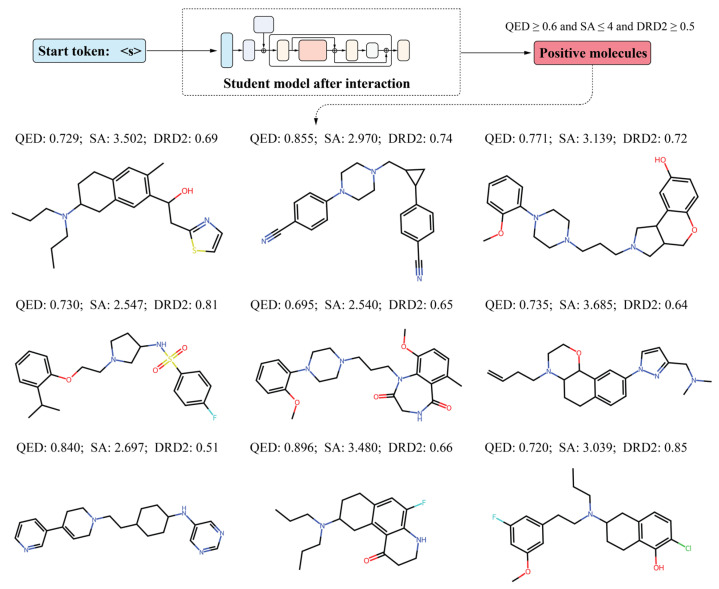
Novel positive molecules randomly generated by the distilled student model. For the DRD2-targeting molecule generation task, nine novel molecules satisfying the DRD2, QED and SA properties were randomly selected and displayed.

**Figure 5:**
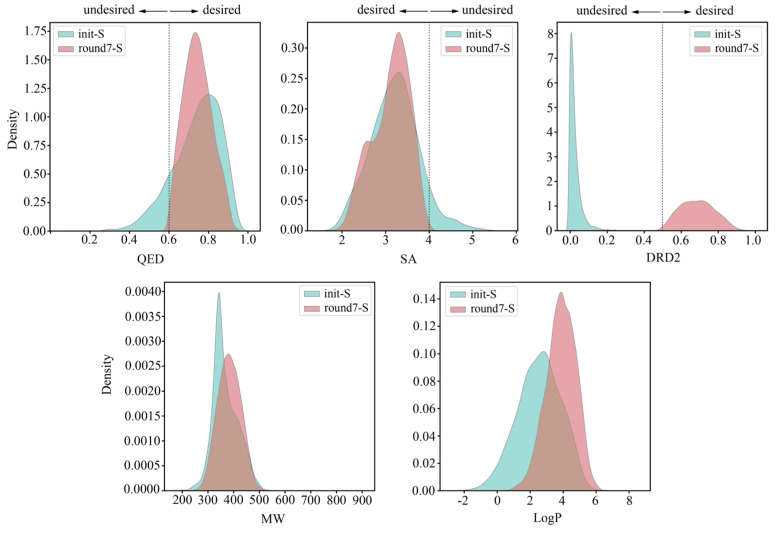
The distributions of the molecular properties for the molecules generated by init-S and round7-S. init-S and round7-S are the student models after the first and seventh rounds of interactive knowledge distillation, respectively.

Notably, unlike previous generative models that require target properties as conditional inputs, the knowledge interaction process enables the student model to directly generate molecules that satisfy multiple properties simultaneously using only the start token <s>. The student model achieved a high benchmark success rate of 0.953 ± 0.001 (Table 3, Figure 4). Random examples of the generated molecules with the DRD2 activity are shown in Figure 4. The randomly generated JNK3/GSK3β-targeting molecules were also displayed in Supplementary Figure S3. We further investigated the distribution of physical, chemical and bioactivity properties of the generated 5000 molecules of the init-S and the student model after seven rounds of interaction, respectively. As shown in Figure 5 and Supplementary Figure S4, the knowledge interaction biased the student model toward the desired multi-property profiles.

**Figure 6:**
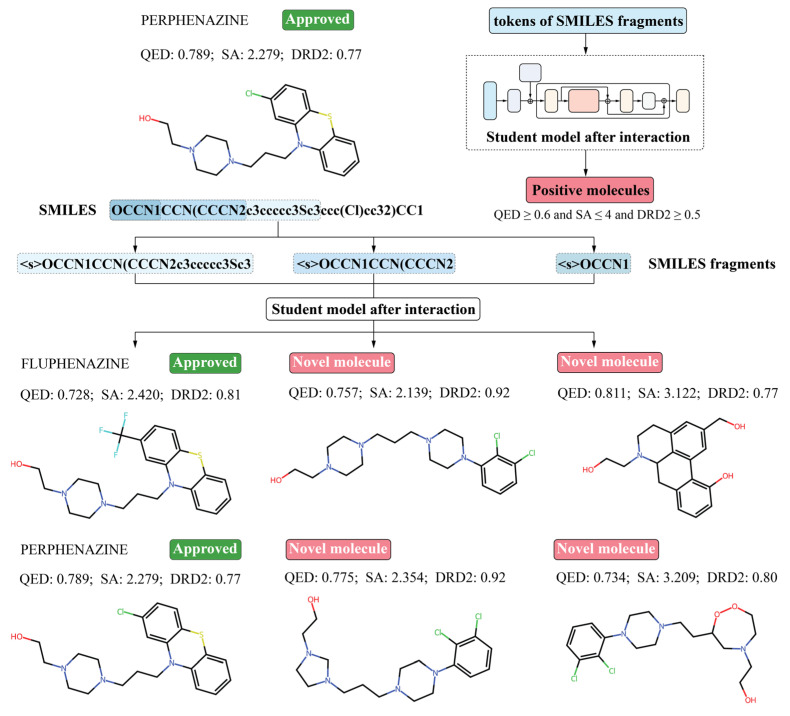
The molecules generated by the distilled student model based on the fragments of the known FDA-approved drug. The drug itself, another FDA-approved drug and other novel molecules satisfying the predefined computational criteria are successfully generated. They may contain chemical liabilities and require further medicinal-chemistry filtering and experimental validation.

The student model can also take SMILES fragments as inputs to predict the remaining molecular components. Using the FDA-approved drug perphenazine as an example, we randomly split its SMILES into fragments and used three as inputs. As shown in Figure 6, the student model generated molecules satisfying the predefined computational DRD2 activity, QED, and SA criteria, including perphenazine itself and another FDA-approved drug, fluphenazine. When different SMILES fragments were used as inputs, the model generated structurally varied molecules with predicted property scores that satisfied the benchmark criteria. These examples are intended to illustrate the fragment-conditioned generation capability of the model. They should not be interpreted as experimentally validated molecules or directly developable candidates, because additional chemical-liability filtering, a medicinal-chemistry inspection, a synthesis assessment, and biological validation are required. In particular, some generated molecules may contain reactive or unstable motifs that are not fully captured by the QED and SA.

### 2.4. Student Model Readjustment by RL System

**Figure 7:**
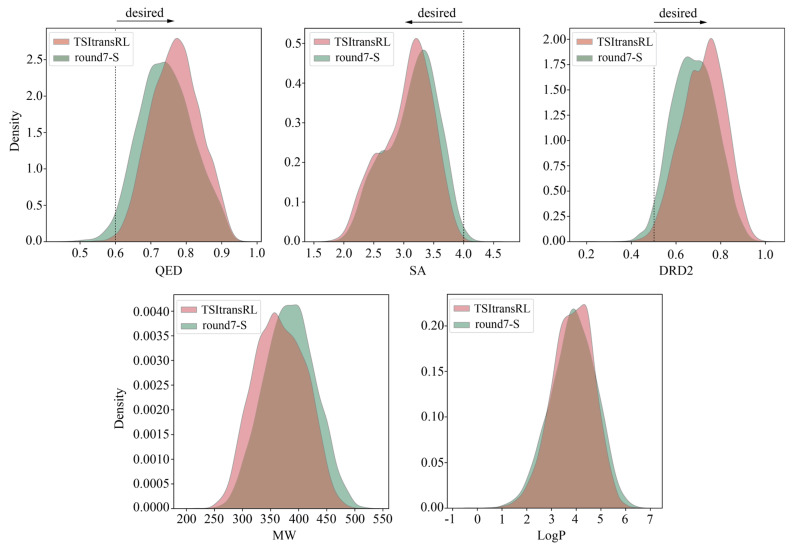
Distributions of molecular properties for molecules generated by the Round-7 student model and TSItransRL. TSItransRL denotes the Round-7 student model further fine-tuned by reinforcement learning.

Reinforcement learning (RL) has been widely used in molecule optimization tasks to guide generative models toward molecules with desired computational properties. As the success rate of the student model gradually reached a plateau during knowledge interaction, we readjusted the student model using RL (TSItransRL) to further improve the performance. We designed an RL system that optimizes the student model through two aspects (Figure 1d): a reward model that evaluates molecular properties and KL divergence, which measures the dissimilarity between probability distributions (see details in Section 4). Their comprehensive score was utilized as a reward to update the student model using proximal policy optimization, guiding the model to generate more successful molecules while avoiding knowledge forgetting. As shown in Table 4 and Supplementary Table S3, TSItransRL achieved a better benchmark-level performance under the predefined computational evaluation criteria, outperforming most baseline methods in several metrics, with a comparable performance for the SNN metric. The property distribution comparison between TSItransRL and the S-model after the knowledge interaction (Figure 7, Supplementary Figure S5) shows that TSItransRL exhibits slightly more favorable distributions toward molecules satisfying the predefined multi-property criteria. The relatively small difference suggests that the student model after interactive knowledge distillation had already learned a favorable multi-property distribution, leaving limited room for further improvement by RL. Therefore, in this benchmark, RL mainly functions as a final fine-tuning step to further bias generation toward the predefined reward criteria.

### 2.5. Exploratory Docking Analysis for Preliminary In Silico Prioritization

**Figure 8:**
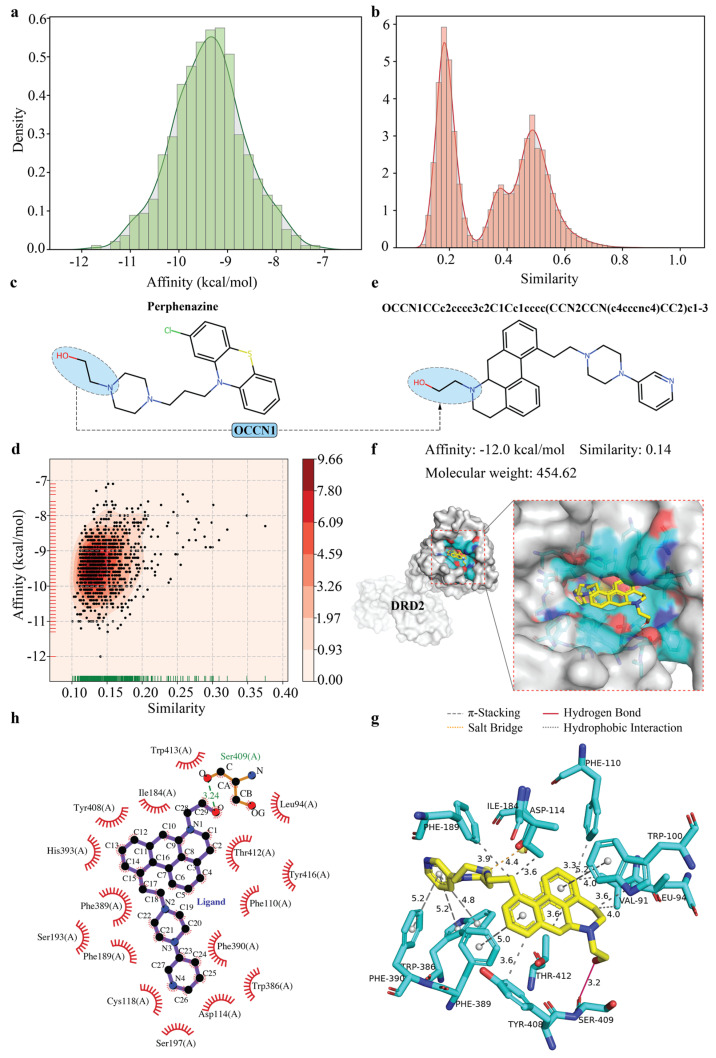
Exploratory docking analysis. (**a**) Distribution of predicted Vina docking scores for 5000 molecules generated by TSItransRL using SMILES fragments of perphenazine as input. (**b**) Distribution of the internal Tanimoto similarity for these molecules. (**c**) Example of fragment-based molecular generation from the perphenazine template. (**d**) Relationship between predicted docking score and external similarity to perphenazine. (**e**) The generated molecule with the lowest affinity score. (**f**–**h**) Predicted docking pose of the generated molecule with the most favorable docking score in this exploratory analysis.

To provide a qualitative and exploratory visualization of possible ligand–receptor binding poses, we performed a molecular docking analysis using the DRD2 task as an example. This analysis was intended only for preliminary in silico prioritization and should not be interpreted as validation of the binding affinity, target engagement, or biological activity. Using the approved drug perphenazine (Figure 8c) as a template, we input its SMILES fragment (for instance, OCCN1) into TSItransRL to generate 5000 molecules. Docking was performed using AutoDock Vina v.1.2.2, and PyMOL v.3.1.4 was used to visualize the predicted binding poses. Perphenazine was docked using the same workflow and served only as an internal computational reference for the predicted docking-score and binding-pose inspection.

The distribution of predicted docking scores is shown in Figure 8a. Several generated molecules showed lower predicted Vina scores than perphenazine under the same computational workflow. However, the Vina scores are approximate scoring-function outputs and should not be interpreted as quantitative binding free energies or evidence of an improved affinity. The internal similarity distribution of the generated molecules (Figure 8b) showed an average Tanimoto similarity of 0.35, suggesting that the generated molecules retained structural diversity. An external similarity analysis relative to perphenazine (Figure 8d) further indicated that many of the generated molecules had a relatively low similarity to perphenazine while showing favorable predicted docking scores in this exploratory analysis. These observations suggest that some generated molecules may be selected for further computational inspection, but they do not establish improved receptor interaction profiles or biological activity.

For qualitative visualization, the generated molecule with the most favorable predicted docking score in this exploratory analysis was selected as a representative example (Figure 8f–h). The predicted docking pose suggested possible contacts with the DRD2 binding region, including hydrogen-bonding and hydrophobic contacts. These observations should be interpreted only as exploratory computational visualizations rather than evidence of DRD2 binding, an improved affinity, or biological activity. Experimental biochemical or cell-based assays are required to confirm the actual binding affinity and functional activity. Docking analyses for the JNK3/GSK3β task are provided in Supplementary Figures S5–S7.

The present docking analysis was not designed as a validated structure-based virtual-screening workflow. A complete docking validation procedure, including detailed receptor-preparation parameters, a ligand protonation-state assessment, docking-box validation, redocking of co-crystallized ligands with RMSD reporting, known ligand/decoy benchmarking, a ligand-efficiency-normalized comparison, and experimental binding assays, was not performed. Therefore, the docking results should be regarded only as exploratory visualizations and preliminary computational prioritization evidence.

### 2.6. Similarity Ensemble Approach (SEA) Analysis

**Figure 9:**
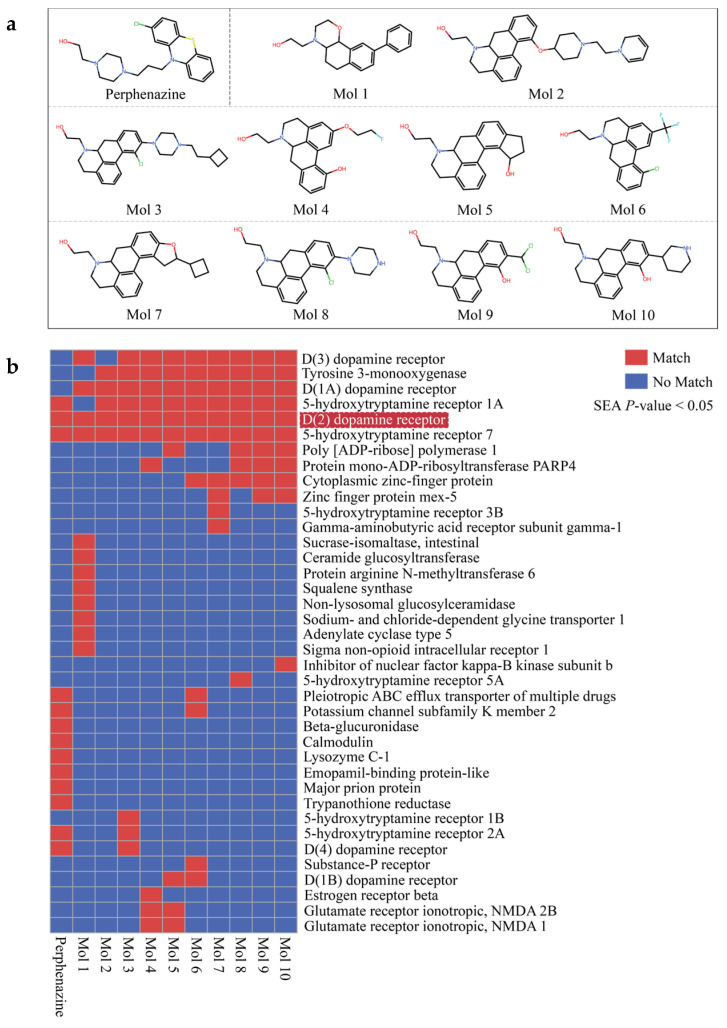
The SEA analysis for the molecules generated by TSItransRL. (**a**) Ten molecules randomly sampled from the novel molecules generated by TSItransRL. (**b**) The heatmap of the molecules and the potential targets.

We analyzed the potential receptor targets of molecules generated by TSItransRL in the DRD2-targeting task using the SEA, a cheminformatics approach that uses drug chemistry similarity to identify targets for small molecules. In this analysis, molecules were represented using ECFP4/Morgan circular fingerprints, and the pairwise molecular similarity was calculated using the Tanimoto coefficient. Target associations were inferred by comparing the generated molecules with known ligand sets of potential targets, and statistically significant associations were identified using SEA *p*-values. Ten molecules were randomly sampled from successful molecules (Mol 1–10, Figure 9a) and their potential targets were obtained. After filtering by the SEA *p*-value (<0.05), the top 15 significant receptor targets were selected. As shown in Figure 9b, the D(2) dopamine receptor (DRD2) was identified among the significant targets, supporting the potential association of the generated molecules with the desired target class. Several other potential receptor targets were found, including dopamine receptors (D1, D3, D4), tyrosin 3-monooxygenase, and 5-hydroxytryptamine receptors, primarily neurological receptors and enzymes involved in pharmaceutical targets for nervous system disorders. These results suggest that the generated molecules preserve chemical features associated with dopaminergic and other neurological target classes, which is consistent with the perphenazine-derived fragment input.

**Figure 10:**
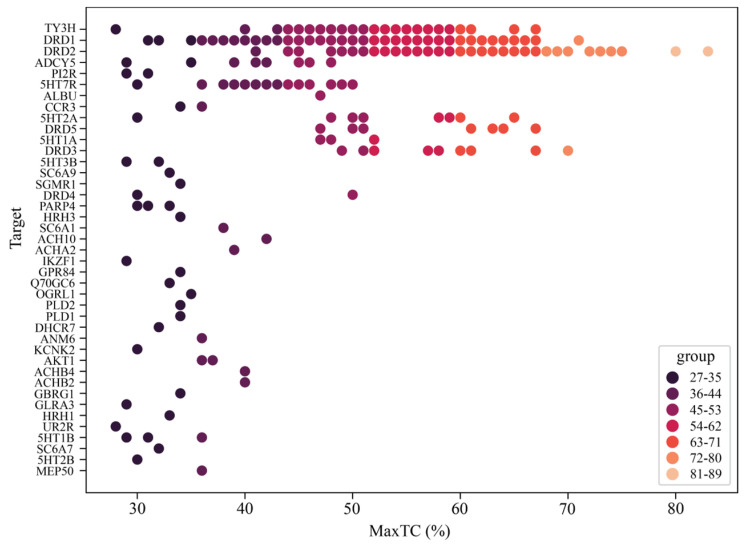
The maximum Tanimoto coefficient (MaxTC) between the novel DRD2-targeting molecules generated by TSItransRL and the known ligands in ChEMBL for a given target.

We further calculated the maximum Tanimoto coefficient (MaxTC) between novel molecules and known ligands in ChEMBL for a given target. As illustrated in Figure 10, all of the sampled molecules showed MaxTC > 0.4 for the DRD2 target, indicating chemical similarity to known DRD2 ligands. Over 92% of the generated DRD2-targeting molecules had a MaxTC > 0.5 (ECFP4), suggesting that they may be prioritized for further computational inspection and possible experimental evaluation according to ligand-similarity-based criteria [31]. The SEA analysis for the supplementary dual JNK3/GSK3β-targeting benchmark is provided in Supplementary Figure S8.

### 2.7. Scaffold Analysis of the Generated Molecules

**Figure 11:**
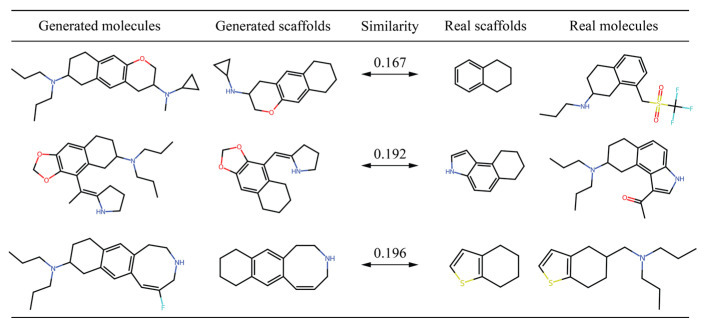
The top differential scaffolds of the DRD2-targeting molecules generated by TSItransRL. The first column shows the molecules generated by TSItransRL, the second column shows the scaffolds of these molecules, the third column shows the similarity between the scaffolds of the generated molecules and the real molecules, and the fourth and fifth columns show the scaffolds of the molecules from the real activity dataset and the real molecules, respectively.

We performed a scaffold analysis on 10,000 molecules generated by different models in the DRD2-targeting task (task 1). The generated molecules were first canonicalized, and molecules without valid Murcko scaffolds were excluded from the scaffold-similarity analysis. For comparison with previously published baseline models, the scaffold similarity results for REINVENT, REINVENT2.0, MCMGL, MCMGM, and semi-MCMGM were taken from Wang et al. [10]. Because the original generated molecule sets for these baseline models were not available, additional scaffold statistics, including unique scaffold counts and normalized percentages, were newly calculated for TSItransRL. For TSItransRL, 9621 generated molecules with valid Murcko scaffolds were analyzed, corresponding to 3575 unique Murcko scaffolds. Each generated molecule was assigned to a scaffold-similarity interval based on the maximum Tanimoto similarity between its Murcko scaffold and the reference active scaffold set. The normalized percentages across the intervals ≤0.1, 0.1~0.2, 0.2~0.3, and 0.3~0.4 were 0.00%, 0.17%, 12.37%, and 12.03%, respectively. To keep the main comparison consistent with previously reported baseline results, Table 5 summarizes the number of generated molecules falling into the scaffold-similarity intervals of ≤0.1, 0.1~0.2, 0.2~0.3, and 0.3~0.4, together with the average pairwise scaffold similarity. Following Wang et al., the average pairwise scaffold similarity was calculated as the mean of all pairwise Tanimoto similarities between the Murcko scaffold of each generated molecule and the Murcko scaffolds of all active reference molecules. TSItransRL achieved an average pairwise scaffold similarity of 0.131, lower than the baseline values reported by Wang et al., suggesting that the generated molecules explored a scaffold space distinct from the known active molecules at the global pairwise-similarity level. Representative generated candidates with a low scaffold similarity are visualized in Figure 11. The improvement of TSItransRL was also observed for task 2 in Supplementary Table S4 and Figure S9.

### 2.8. Web Application of TSItransRL

**Figure 12:**
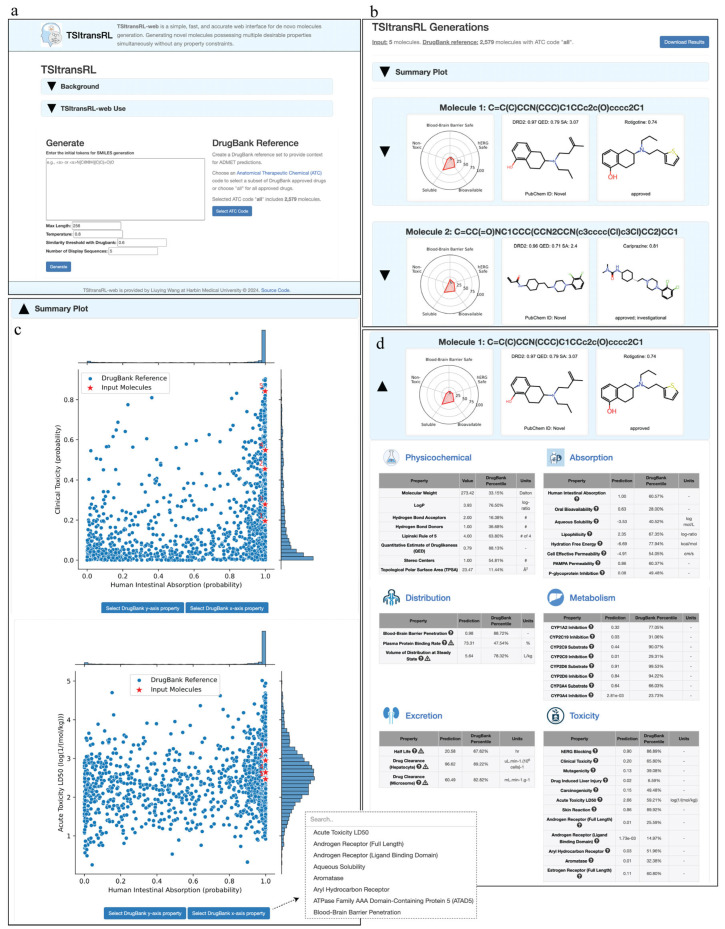
**The TSItransRL-web interface.** (**a**) The main interface, in which start tokens or partial SMILES tokens are required. (**b**) The radial plot summarizing five key ADMET properties of the generated molecule (**left**). The generated molecule satisfying predefined predicted DRD2 activity, QED, and SA criteria (**middle**). The most similar drug in DrugBank for the generated molecule (**right**). (**c**) The distribution plot of ADMET properties for the top molecules of all the generated molecules compared to the DrugBank reference set. (**d**) The physicochemical and ADMET properties of the displayed molecule generated by TSItransRL.

To provide a user-facing implementation of the proposed computational workflow, we developed a web application (TSItransRL-web) based on TSItransRL. Using the DRD2 task as an example, TSItransRL-web can generate molecules satisfying the predefined predicted DRD2 activity, QED, and SA criteria using only a start token or partial SMILES tokens as input (Figure 12a). The application displays generated molecules ranked by the predicted DRD2 activity among 5000 generated candidates (Figure 12b, middle panel) and compares their similarity with DrugBank reference drugs, showing the most similar approved, investigational, or experimental DRD2-targeting drugs in the right panel. This provides reference information for preliminary computational prioritization and illustrates the ability of TSItransRL-web to generate molecules satisfying predefined predicted-property criteria.

For additional exploratory profiling, TSItransRL-web employs ADMET-AI [32] to assess the absorption, distribution, metabolism, excretion, and toxicity (ADMET) properties. The summary plot (Figure 12c) shows the ADMET property distribution for the top molecules compared to the DrugBank reference set, demonstrating that the generated molecules were predicted to have intestinal absorption and acute toxicity profiles within ranges comparable to those of approved drugs in the reference set. Each molecule features a radial plot summarizing five key ADMET properties in DrugBank percentiles (Figure 12b, left panel). The platform calculates eight physicochemical properties using RDKit and 41 ADMET properties using the Chemprop-RDKit graph neural networks, with detailed tabular results accessible for each molecule (Figure 12d). Overall, TSItransRL-web can serve as a useful tool for the preliminary in silico profiling of generated molecules with a specified predicted target bioactivity, while further validation is required before any practical drug-discovery application.

## 3. Discussion

In this study, we developed TSItransRL, a novel teacher–student interaction transformer combined with reinforcement learning for in silico multi-property molecular generation. The framework achieved success rates of 98.36% for the DRD2-targeting benchmark and 98.90% for the dual JNK3/GSK3β-targeting benchmark under predefined computational criteria. These findings support TSItransRL as a computational proof of concept for benchmark-level multi-property molecular generation. However, the results are based on the predicted target activity and QED/SA constraints and should not be interpreted as evidence of experimentally validated biological activity, lead identification, or practical drug-discovery utility.

The evolution of computer-aided drug design (CADD) over the past decades has increasingly focused on the critical challenge of multi-property optimization, which represents one of the most significant bottlenecks in contemporary drug discovery. Traditional CADD approaches, including structure-based drug design (SBDD) and ligand-based drug design (LBDD), typically optimize single properties or require manual balancing of multiple objectives through iterative design cycles. There is increasing interest in computational methods that can jointly consider the target activity, selectivity-related information, ADMET-related properties, and synthetic accessibility. Our TSItransRL framework addresses this computational challenge by generating molecules that satisfy multiple predefined benchmark constraints without requiring explicit conditional inputs during inference, providing an alternative strategy for integrated multi-property molecular generation.

The knowledge interaction mechanism specifically tackles the multi-property optimization challenge through progressive knowledge transfer between teacher and student models. Traditional data-driven generative models struggle with the scarcity of molecules possessing multiple desirable characteristics simultaneously, as demonstrated by our datasets where positive molecules comprised only 6.7–7.2% of the total compounds. This data sparsity problem becomes exponentially more severe when multiple properties must be satisfied concurrently, as the intersection of molecules meeting all criteria shrinks dramatically. The teacher–student interaction enables the student model to learn from an expanding repository of multi-property positive molecules generated by the teacher model, effectively addressing the data scarcity issue that has historically limited multi-property drug-design efforts. This iterative process resulted in a dramatic improvement in success rates from 0% (initial student model) to 89.5% (after interaction), demonstrating the effectiveness of this collaborative learning strategy for multi-property molecular generation.

The improved benchmark performance compared with baseline methods suggests that interactive knowledge distillation may provide useful guidance for multi-property molecular generation under the evaluated computational criteria. Unlike existing methods that require users to specify property ranges for each desired characteristic—often leading to conflicting constraints and invalid molecule generation—TSItransRL generates molecules with multiple desired properties using only start tokens. This approach eliminates the complex parameter tuning required for balancing multiple objectives and reduces the risk of generating molecules that satisfy some properties while failing others. The ability to achieve high success rates across multiple predefined computational criteria indicates an improved benchmark performance relative to several evaluated baselines.

The integration of reinforcement learning with KL divergence as part of the reward function specifically addresses the multi-property optimization challenge by preventing mode collapse while simultaneously optimizing multiple target characteristics. By maintaining distributional similarity to the pre-fine-tuned model while optimizing target properties, TSItransRL prevents convergence to narrow chemical space regions that might satisfy one property at the expense of others. This strategy effectively balances property optimization with molecular diversity, as evidenced by the high internal diversity (0.795) and novelty (0.998) scores achieved while maintaining high success rates across multiple property dimensions. The exploratory docking analysis was used only for qualitative visualization and preliminary prioritization. Although some generated molecules showed favorable predicted docking scores and binding poses under the same workflow, the Vina scores are approximate scoring-function outputs and cannot establish an improved binding affinity, target engagement, or biological activity. Instead, they provide only exploratory computational context alongside the predicted activity, SEA/MaxTC, scaffold, and ADMET analyses.

Mode collapse was further mitigated through mechanisms in both the interactive knowledge distillation and reinforcement learning stages. During knowledge distillation, the student model was pretrained on PubChem-10 M, which provided a broad chemical-space prior before target-specific optimization. The teacher–student interaction was also bidirectional: the teacher generated positive pseudo-samples under the condition label c = 1 to guide the student model, whereas the student generated molecules without explicit condition labels from the broader PubChem-derived distribution, and these molecules were evaluated, labeled, and fed back to update the teacher model. This iterative feedback reduced the risk that both models would converge to a narrow subset of high-scoring molecules. During reinforcement learning, KL divergence regularization in PPO penalized excessive deviation of the updated student model from the frozen pre-fine-tuned reference model, thereby encouraging reward improvement while preserving molecular diversity. The high internal diversity, novelty, and scaffold novelty observed for TSItransRL indicate that the model maintained broad chemical-space exploration rather than collapsing into a limited set of molecular structures.

The modest improvement introduced by reinforcement learning over the Round-7 student model should be interpreted in the context of the staged optimization strategy of TSItransRL. The interactive knowledge distillation stage already guides the student model toward the desired multi-property molecular distribution, resulting in a strong performance before reinforcement learning. Thus, in the current benchmark, RL is not the dominant source of improvement, but serves as a flexible reward-driven refinement component after interactive knowledge distillation. Its contribution is to further bias generation toward high-reward molecules and to provide a mechanism for incorporating additional or more stringent property objectives. The limited gain observed here is therefore likely due to diminishing returns after the strong Round-7 student model performance, rather than indicating that RL is generally unnecessary. In more challenging tasks, or when the post-interaction student model has not fully converged to the desired region, the RL component may provide larger benefits.

The comparison with RL-based baselines further supports the contribution of the teacher–student interaction strategy. REINVENT and REINVENT2.0 optimize molecular generation through reinforcement learning without using the teacher–student interaction or interactive knowledge distillation. The superior performance of TSItransRL over these baselines indicates that the proposed interaction mechanism provides effective target-specific guidance before RL fine-tuning. Moreover, the staged performance within TSItransRL provides an ablation-style interpretation: the initial student model generated almost no successful molecules, whereas the student model after the initial teacher–student interaction achieved a success rate of 89.5%, and the Round-7 student model reached 95.3%. RL fine-tuning further improved the success rate to 98.36%. These results suggest that interactive knowledge distillation is the primary contributor to learning the desired multi-property distribution, while RL provides additional reward-driven refinement.

Within the broader CADD context, TSItransRL may be viewed as a computational framework for exploring molecules that satisfy multiple predefined predicted-property constraints. Unlike sequential single-property optimization workflows, the proposed approach jointly considers the predicted activity, QED, and SA criteria in the benchmark setting. However, whether this strategy improves real lead-optimization workflows remains to be determined through independent validation, prospective testing, and experimental assessments. While traditional approaches such as molecular docking and QSAR modeling excel at predicting or optimizing individual properties, they typically require sequential application and manual integration to address multiple objectives. The present results indicate that TSItransRL can jointly optimize several predefined computational criteria in the evaluated benchmarks. However, broader claims regarding advantages over real-world lead-optimization workflows require prospective comparison and experimental validation.

The SEA and MaxTC analyses provided supportive ligand-similarity-based evidence that many generated molecules are chemically associated with the desired target class. For the DRD2 task, a large proportion of generated molecules showed relatively high MaxTC values to known DRD2 ligands, suggesting that they preserve chemical features commonly found in DRD2-active compounds. However, the SEA and MaxTC analyses do not directly demonstrate target specificity, selective binding, or functional activity. Because several generated molecules were also associated with other neurological receptors and enzymes, including dopamine receptor subtypes, 5-hydroxytryptamine receptors, tyrosine hydroxylase, and adenylate cyclase-related targets, these associations may indicate either potentially useful polypharmacology or possible off-target liabilities. Therefore, the predicted target-association profiles should be interpreted as a prioritization tool rather than definitive evidence of selectivity. Experimental selectivity profiling across relevant receptor panels will be necessary to distinguish desired target engagement from undesirable off-target effects.

The current results should not be taken as evidence that TSItransRL can reduce the drug-development cost, shorten development timelines, or reduce design–make–test cycles. At this stage, the framework is best regarded as a computational prioritization method under predefined benchmark criteria. If combined with rigorous external validation, experimental testing, and medicinal-chemistry optimization, such a framework may help identify molecules for further evaluation, but this practical utility remains to be demonstrated.

The current implementation of TSItransRL requires target-specific bioactivity information to construct binary condition labels for teacher-model pre-training. In this study, the teacher model was trained using 7219 positive and 100,000 negative molecules for the DRD2 task, and 3405 positive and 100,000 negative/background molecules for the JNK3/GSK3β task, which were sufficient to support stable teacher–student interactions and a high generation performance. Empirically, these results suggest that several thousand positive molecules, together with a sufficiently large negative/background set, can support effective teacher-model pre-training. However, this should be interpreted as an empirical observation from the current tasks rather than a universal minimum requirement. For low-data targets, TSItransRL may be extended by using transfer learning from related targets, multitask activity predictors, pretrained QSAR models, target-family-level datasets, or active learning to construct approximate condition labels. Nevertheless, when neither sufficient bioactivity data nor reliable predictive models are available, teacher-model pre-training may become less reliable and the generation performance may decrease. Future studies should systematically evaluate the sensitivity of TSItransRL to the dataset size and label quality, especially in low-data target scenarios.

Despite the promising computational results, several important limitations warrant consideration. Although the target-specific datasets and task definitions followed the established benchmark protocol of Wang et al. [10] rather than being defined de novo by our reward model, the primary success metrics were still based on the predicted target activity and predefined QED/SA thresholds. Therefore, the high success rates should be interpreted as evidence that TSItransRL efficiently optimizes established computational benchmark criteria, rather than as proof of experimentally confirmed biological activity or developability. The present study does not provide experimental validation of biological activity, target selectivity, chemical stability, toxicity, or synthetic feasibility. Independent validation using external QSAR models, scaffold/time-split evaluations, known active/decoy comparisons, and, ultimately, biochemical or cell-based assays will be required to confirm the true biological activity and selectivity of the generated molecules. Second, the QED and SA are simplified heuristic metrics and do not capture many critical medicinal-chemistry liabilities. Generated molecules may still contain chemically unstable, reactive, metabolically labile, or assay-interfering substructures, even if they satisfy QED and SA thresholds. Therefore, additional filters and evaluations, such as PAINS and toxicophore screening, reactive functional-group alerts, and expert medicinal-chemistry inspection, should be incorporated before selecting molecules for synthesis or biological testing. Third, the computational requirements for teacher–student interactions and RL fine-tuning may limit the accessibility for resource-constrained research environments. Although REINVENT and REINVENT2.0 provide relevant RL-based comparisons, an architecture-matched RL-only ablation, in which the PubChem-pretrained student model is directly optimized by RL without teacher–student interactions, would provide a more controlled quantification of the contribution of interactive knowledge distillation and should be considered in future work.

Future research directions should focus on expanding the scope and sophistication of multi-property optimization capabilities. The integration of additional property dimensions, such as more detailed synthetic-feasibility estimates, chemical-liability filters, ADMET-related predictors, and target-selectivity models, could create more comprehensive computational evaluation frameworks. The development of active learning protocols that incorporate experimental feedback could help evaluate and refine the model in prospective molecular design settings. Furthermore, extending the framework to additional molecular design tasks may help evaluate whether TSItransRL can contribute to future experimentally informed molecular generation workflows.

In summary, TSItransRL provides an in silico framework for generating molecules that satisfy predefined computational multi-property criteria while maintaining molecular diversity and scaffold novelty. The results show that interactive knowledge distillation combined with reinforcement-learning refinement can improve the benchmark-level performance for molecular generation under predicted activity, QED, and SA constraints. However, the generated molecules should be regarded as computational candidates for prioritization and hypothesis generation, not experimentally validated leads or drug candidates. Further independent validation using external predictive models, scaffold/time-split evaluations, synthesis assessment, and biochemical or cell-based assays will be required before the practical drug-discovery utility can be established.

## 4. Materials and Methods

### 4.1. Model Architecture of TSItransRL

TSItransRL comprises three main stages (Figure 1): (1) the pre-training of a conditional teacher transformer and vanilla student transformer, (2) interactive knowledge distillation between the teacher and student models, and (3) reinforcement learning fine-tuning of the distilled student model. In the first stage, the teacher model learns the relationship between the molecular structures and predefined multi-property labels from target-specific datasets, whereas the student model learns general SMILES syntax and chemical structure distributions from PubChem-10 M. In the second stage, the teacher model provides positive multi-property pseudo-samples to guide the student model, while the student model feeds back newly explored labeled molecules to update the teacher model. In the final stage, the student model is further optimized by proximal policy optimization (PPO) using property-based rewards and KL regularization.

***Pre-training process.*** The pre-training process is illustrated in Figure 1a. The teacher model was implemented as a conditional transformer and pretrained on target-specific molecular datasets. Each training sample for the teacher model was represented as a pair (SMILES, c), where SMILES denotes the molecular sequence and c is a binary condition label indicating whether the molecule satisfies all predefined task-specific property criteria. The label c was precomputed before training using QED, SA, and target-activity prediction models according to the task definitions in Section 4.2. Molecules satisfying all criteria were assigned c = 1, whereas the remaining molecules were assigned c = 0.

During teacher-model pre-training, the binary label c was converted into a condition token and prepended to the SMILES sequence. The molecule sequence tokens were embedded as $X_{1}$, and the condition token derived from c was embedded as vector $X_{2}$. The combined input embeddings were then encoded by the teacher model as the hidden representation $X_{3}$, which was forwarded into the masked multihead attention layers of the transformer network to extract key sequence features Z. The conditional probability of the next SMILES token was calculated as follows:

(1) $$p_{teacher}\left(x_{j}|x_{<j},c\right)=softmax\left(Z_{x_{<j},c}\right)$$

where $Z_{x_{<j},c}$ is the extracted sequence feature, given the conditional label $c\in \left\{0,1\right\}$ and the preceding SMILES tokens of length j−1 as input. $p_{teacher}\left(x_{j}|x_{<j},c\right)$ is the conditional probability of the *j*th SMILES token. The pre-trained teacher model was optimized by minimizing the following negative log-likelihood with a given conditional label *c.*

(2) $$L_{teacher}\left(\theta \right)=-\sum_{i=1}^{n}\log p_{teacher}\left(\theta ,x_{j}|x_{<j},c\right)$$

Different from the teacher model, the student model was implemented as a vanilla transformer and received only SMILES sequences or sequence tokens without conditional property labels. During pre-training, the student model was trained on PubChem-10 M SMILES strings to model the unconditional probability distribution of valid molecules by minimizing the following negative log-likelihood:

(3) $$L_{student}\left(\theta \right)=-\sum_{j=1}^{n}\log p_{student}\left(\theta ,x_{j}|x_{<j}\right)$$

***Interactive knowledge distillation.*** After pre-training, the teacher and student models were updated through an interactive bidirectional knowledge distillation procedure. The teacher and student generation distributions are defined as follows:

(4) $$p_{teacher}\left(x|c\right)=\prod_{i=1}^{n}p_{teacher}\left(\theta_{teacher},x_{j}|x_{<j},c\right)$$

(5) $$p_{student}\left(x\right)=\prod_{j=1}^{n}p_{student}\left(\theta_{student},x_{j}|x_{<j}\right)$$

In each interaction round, the teacher model first generated candidate molecules under the positive condition label (c = 1). Therefore, molecules were sampled from the conditional distribution $p_{teacher}\left(x|c=1\right)$, which biased generation toward the desired multi-property region. The generated SMILES strings were canonicalized and checked for validity using RDKit v.2024.3.1. Their QED, SA, and target-activity scores were then calculated using the same property evaluation models and task-specific criteria defined in Section 4.2. Only valid molecules satisfying all predefined criteria were retained as positive pseudo-samples and used to fine-tune the student model.

After being fine-tuned with these teacher-generated positive molecules, the student model generated molecules using only the start token <s> or partial SMILES tokens as input, without receiving any explicit condition label. The student-generated molecules were also canonicalized, filtered for validity, and evaluated according to the same predefined criteria. Each valid molecule was assigned a binary label according to its evaluated properties: molecules satisfying all criteria were labeled as c = 1, whereas the remaining valid molecules were labeled as c = 0. The resulting labeled samples, represented as (SMILES, c), were then used to update the teacher model.

This teacher-to-student and student-to-teacher interaction was repeated for seven rounds. Through this process, the teacher model progressively provided positive multi-property molecular examples to guide the student model, while the student model fed back newly explored labeled molecules from the broader PubChem-derived chemical space to refine the teacher model. As a result, the student model gradually acquired the ability to generate molecules satisfying multiple desired properties without requiring explicit condition labels during inference.

***Reinforcement learning fine-tuning.*** To further improve the performance, we fine-tuned the distilled student model using proximal policy optimization [33]. The process involves: (1) generating SMILES sequences from query tokens using two identical student models (one updated, one frozen as reference), (2) evaluating sequences with a customized reward function, and (3) combining reward scores with KL divergence to guide model updates while preventing deviation from the desired property distribution.

### 4.2. Evaluation Settings

Two multi-objective generation tasks were conducted: dopamine type 2 receptor (DRD2)-targeting molecule generation and c-Jun N-terminal kinase-3 (JNK3)/glycogen synthase kinase-3 beta (GSK3β)-targeting molecule generation. The task definitions, including the target-activity thresholds and QED/SA constraints, followed the benchmark settings reported by Wang et al. [10] to ensure consistency with previously published baseline models. These benchmark settings were adopted from prior studies and were not defined using the reward model developed in this work. To avoid ambiguity, we distinguished the raw synthetic accessibility score from the SA reward component used during reinforcement learning. The raw SA score was calculated using RDKit and was used for threshold-based evaluation, where molecules satisfying the predefined SA constraint were considered synthetically accessible under the benchmark criterion. During reinforcement learning, the raw SA score was not directly added as a positive reward term. Instead, it was converted into a binary SA reward component: molecules satisfying the SA constraint received a reward value of 1 for this component, whereas molecules not satisfying the constraint received a value of 0. This transformation ensured that molecules with higher, less desirable raw SA scores were not rewarded. The predicted target-activity score(s) and QED were used directly as reward components because they lie within the range of 0 to 1, and the transformed binary SA reward was also within the same range. No additional task-specific weighting was applied among the property reward components; they were combined as an unweighted sum. In the PPO fine-tuning stage, KL regularization was used separately to limit excessive deviation from the reference student model distribution. The specific task definitions are as follows:

*DRD2-targeting molecule generation (task 1).* The objective was to generate molecules with a predicted DRD2 activity greater than or equal to 0.5, a QED greater than or equal to 0.6, and an SA less than or equal to 4. The QED and SA were calculated using RDKit, and the DRD2 activity was predicted using the predictive model reported by Olivecrona and colleagues [34]. For reinforcement learning, the DRD2 activity score and QED were used directly as continuous reward components, while the raw SA score was converted into the binary SA reward component described above. Therefore, the SA term in the reward function denotes the transformed SA reward component rather than the raw SA score. The reward score function $S\left(m\right)$ for molecule m is shown below:

(6) $$S\left(m\right)=DRD2\left(m\right)+QED\left(m\right)+SA\left(m\right)$$

*JNK3/GSK3*β-*targeting molecule generation (task 2)*. The objective was to generate molecules with predicted JNK3 and GSK3β activities greater than or equal to 0.5, a QED greater than or equal to 0.6, and an SA less than or equal to 4. The activities for JNK3 and GSK3β were obtained using the predictive model in the work by Jin et al. [12]. The reward score function $S\left(m\right)$ for molecule m is shown below:

(7) $$S\left(m\right)=JNK3\left(m\right)+GSK3\beta \left(m\right)+QED\left(m\right)+SA\left(m\right)$$

For comparison, we evaluated TSItransRL against the c-transformer, DL, DM, MCMGL, MCMGM, Semi-MCMGM, REINVENT, and REINVENT2.0. The former six models were trained according to the description in Wang et al. [10], and the latter two models were trained following previously reported settings.

### 4.3. Data Preparation

For teacher-model pre-training, target-specific molecular datasets were used to construct labeled samples. These datasets were obtained from previously published bioactivity benchmark datasets used by Wang et al. [10]. The DRD2 dataset was originally provided by Olivecrona et al. [34] and selected from ChEMBL, containing 7219 positive molecules and 100,000 negative molecules. The JNK3/GSK3β datasets were obtained from the study by Jin et al. [12]; the JNK3 dataset contains inhibition data for 2665 positive and 50,000 negative compounds, whereas the GSK3β dataset contains inhibition data for 740 positive and 50,000 negative compounds. For the dual-target JNK3/GSK3β task, these datasets were combined, resulting in 3405 positive molecules and 100,000 negative molecules. Each molecule was represented as a SMILES sequence and assigned a binary condition label c according to the task-specific property criteria defined in Section 4.2.

For student-model pre-training, 10 million molecules from PubChem-10 M were used as the knowledge base. These molecules were used only as unlabeled SMILES sequences without condition labels, enabling the student model to learn general SMILES syntax and molecular distributions. The datasets were split into training and test sets at a ratio of 9:1. During interactive knowledge distillation, seven rounds of teacher–student interactions were performed. In each round, 500,000 candidate molecules were sampled from the teacher model conditioned on c = 1, and valid molecules satisfying all task-specific criteria were selected as positive pseudo-samples to fine-tune the student model. The updated student model then generated molecules without condition labels. These molecules were evaluated using the same criteria and assigned binary labels before being used to further update the teacher model. For reinforcement learning fine-tuning, 500,000 generated molecules with start tokens or partial SMILES fragments as inputs were used to optimize the distilled student model.

### 4.4. Hardware and Experiment Settings

All the experiments were performed using a workstation equipped with two NVIDIA Quadro GV100 32 GB GPU cards. We implemented the model using the PyTorch framework (version 1.10). The molecular docking was conducted using AutoDock Vina and was used only as an exploratory visualization and preliminary in silico prioritization analysis. The docking workflow was not intended as a fully validated structure-based virtual-screening protocol. All the plots were made using the matplotlib package (v.3.11) in Python.

For the pre-training process, the model parameters were updated via the Adam optimizer and trained for 100 epochs with a batch size of 128. During the interactive knowledge distillation, we used the SGD optimizer with a learning rate of $5\times 10^{-4}$ to update the model parameters, which was trained for 10 epochs with a batch size of 32. For the reinforcement learning fine-tuning, the distilled student model was trained through proximal policy optimization (PPO) for 30,000 iterations with a batch size of 256 and a learning rate of $1\times 10^{-6}$.

### 4.5. Evaluation Criteria

Following Wang et al. [10], two different sets of metrics were used in our study, namely the standard metrics proposed by Jin et al. [12] and the well-established MOSES metrics.

Conditional metrics. Success: the percentage of molecules generated by the model that meet the multiple molecular properties. Real success: the percentage of unique molecules generated by the model that meet the multiple molecular properties.

Diversity (Div): obtained based on the Tanimoto distance sim (*X*, *Y*) with respect to the Morgan fingerprints of a pair of successful molecules. The equation is as follows:

(8) $$diversity=1-\frac{2}{N_{positive}\left(N_{positive}-1\right)}\sum_{X,Y}sim\left(X,Y\right)$$

where $N_{positive}$ is the number of successful molecules.

MOSES metrics. Validity: the percentage of valid molecules in the generated molecules. Uniqueness: the percentage of the unique molecules generated. Novelty: the proportion of generated molecules that are not in the training set. Internal diversity (IntDiv): based on the average Tanimoto coefficient between each pair of molecules in the generated molecular set:

(9) $$IntDiv\left(G\right)=1-\frac{1}{{\left|set\left(G\right)\right|}^{2}}\sum_{\left(a,b\right)\in set\left(G\right)}TC\left(m_{a},m_{b}\right)$$

where a and b represents any pair of molecules in the generated molecular set G, $m_{a}$ and $m_{b}$ is their Morgan fingerprint.

Fragment similarity (Frag): used to measure how frequently various molecular fragments appear in the generated molecular set G and training set T:

(10) $$Frag\left(G,T\right)=1-\cos\left(f_{G},f_{T}\right)$$

where $f_{G},f_{T}$ are the fragment frequencies, which are calculated using the BRICS function in RDKit.

Nearest neighbor similarity (SNN): the average Tanimoto similarity between a generated molecule $m_{G}$ and its nearest neighbor $m_{T}$ in the training set:

(11) $$SNN\left(G,T\right)=\frac{1}{\left|G\right|}\sum_{\begin{array}{l} m_{G}\in G\\ m_{T}\in T \end{array}}\max TS\left(m_{G},m_{T}\right)$$

## Figures and Tables

**Table 1:** The distribution of positive and negative molecules in the target-specific benchmark datasets used for teacher-model pre-training.

Dataset	Positive Molecules	Negative Molecules
DRD2	7219	100,000
JNK3/GSK3β	3405	100,000

**Table 2:** The MOSES metrics of the 5000 molecules generated by init-TS, init-T, init-S and the state-of-the-art model.↑ indicates that higher values are better, whereas ↓ indicates that lower values are better.

Model	Validity ↑	SNN ↓	Frag ↑	Novelty ↑	IntDiv ↑	Success ↑
c-Transformer	0.904	0.427	0.774	0.993	0.830	25.4%
DL	0.813	0.432	0.605	0.993	0.718	23.6%
DM	0.886	0.434	0.832	0.983	0.835	4.9%
init-T	0.860	0.469	0.954	0.971	0.852	17.0%
init-S	1.000	0.285	0.647	1.000	0.847	0.0%
init-TS	0.997	0.577	0.782	0.995	0.820	89.5%

**Table 3:** The MOSES metrics of the 5000 molecules generated by the teacher model (T-model) and student model (S-model) during the knowledge interaction process. ↑ indicates that higher values are better, whereas ↓ indicates that lower values are better.

Model	Round	Validity ↑	SNN ↓	Novelty ↑	IntDiv ↑	Success ↑
T-model	1	0.861 ± 0.004	0.466 ± 0.002	0.971 ± 0.002	0.853 ± 0.001	0.171 ± 0.003
	2	0.981 ± 0.002	0.575 ± 0.001	0.993 ± 0.001	0.824 ± 0.001	0.784 ± 0.001
	3	0.979 ± 0.0002	0.563 ± 0.003	0.997 ± 0.001	0.823 ± 0.001	0.837 ± 0.001
	4	0.985 ± 0.002	0.552 ± 0.001	0.999 ± 0.001	0.823 ± 0.001	0.865 ± 0.005
	5	0.988 ± 0.001	0.545 ± 0.001	0.999 ± 0.001	0.822 ± 0.001	0.888 ± 0.005
	6	0.987 ± 0.003	0.539 ± 0.001	0.999 ± 0.001	0.821 ± 0.001	0.895 ± 0.0004
	7	0.990 ± 0.001	0.533 ± 0.001	0.999 ± 0.001	0.820 ± 0.001	0.906 ± 0.005
S-model	1	1.000 ± 0.000	0.285 ± 0.001	1.000 ± 0.000	0.847 ± 0.0006	0.000 ± 0.000
	2	0.997 ± 0.0006	0.580 ± 0.002	0.995 ± 0.000	0.821 ± 0.000	0.897 ± 0.005
	3	0.997 ± 0.002	0.570 ± 0.0010	0.998 ± 0.001	0.817 ± 0.001	0.924 ± 0.003
	4	0.998 ± 0.000	0.556 ± 0.002	0.999 ± 0.0006	0.813 ± 0.001	0.934 ± 0.004
	5	0.998 ± 0.0006	0.546 ± 0.0006	0.999 ± 0.0006	0.809 ± 0.000	0.938 ± 0.002
	6	0.999 ± 0.0006	0.540 ± 0.002	0.999 ± 0.0006	0.807 ± 0.001	0.951 ± 0.002
	7	0.999 ± 0.0006	0.536 ± 0.0006	1.000 ± 0.000	0.805 ± 0.0006	0.953 ± 0.001

**Table 4:** The conditional and MOSES evaluation metrics of the successful molecules generated by the TSItransRL and prior arts. ↑ indicates that higher values are better, whereas ↓ indicates that lower values are better.

	Models	Reinvent	Reinvent2.0	MCMGL	MCMGM	Semi-MCMGM	TSItransRL
Conditional metrics	Success ↑	79.46%	91.10%	99.62%	91.83%	94.18%	98.36%
	Div ↑	0.718	0.559	0.587	0.751	0.714	0.849
	Real success ↑	72.8%	72.6%	15.2%	89.26%	82.69%	97.14%
MOSES metrics	Unique ↑	0.916	0.797	0.153	0.972	0.878	0.998
	Frag ↑	0.448	0.394	0.046	0.334	0.214	0.576
	SNN ↓	0.559	0.570	0.505	0.541	0.525	0.564
	IntDiv ↑	0.620	0.517	0.479	0.668	0.624	0.795
	Novelty ↑	0.993	0.987	0.975	0.992	0.992	0.998

**Table 5:** Scaffold similarity metrics for the scaffolds of real active molecules and generated molecules.

Models	Scaffold Similarity				
	≤0.1 ^a^	0.1~0.2 ^a^	0.2~0.3 ^a^	0.3~0.4 ^a^	Average Similarity ^b^
REINVENT	0	0	6	70	0.550
REINVENT2.0	0	0	2	13	0.600
MCMGL	0	1	31	59	0.574
MCMGM	0	2	143	372	0.542
semi-MCMGM	0	13	506	1086	0.446
TSItransRL	0	16	1190	1157	0.131

^a^ Number of generated molecules whose Murcko scaffold maximum similarity to the reference active scaffold set falls within the corresponding similarity interval. ^b^ Mean of all pairwise Tanimoto similarities between the Murcko scaffold of each generated molecule and the Murcko scaffolds of all reference active molecules.

## Data Availability

The data presented in this study are openly available in: The PubChem-10 M dataset was used for student-model pre-training. The target-specific bioactivity datasets used for teacher-model pre-training were obtained from the studies by Olivecrona et al. [34] and Jin et al. [12] and were prepared as described by Wang et al. [10]. These datasets correspond to different protein targets, namely DRD2, JNK3, and GSK3β, and include the reported positive and negative compounds for each target. The code of TSItransRL used in this study is publicly available at https://github.com/liuyingying95/TSItransRL (1 June 2026). The Web application is currently publicly accessible at https://hz-t3.matpool.com:27682/?token=dYTBCZxrnb (1 June 2026).

## Supplementary Materials

The following supporting information can be downloaded at: https://www.mdpi.com/article/10.3390/ijms27146125/s1.

## Abbreviations

The following abbreviations are used in this manuscript:

**TSItransRL**	Teacher–Student Interaction Transformer Fine-Tuned by Reinforcement Learning
**RL**	Reinforcement Learning
**PPO**	Proximal Policy Optimization
**KL Divergence**	Kullback–Leibler Divergence
**SMILES**	Simplified Molecular-Input Line-Entry System
**DRD2**	Dopamine Type 2 Receptor
**JNK3**	c-Jun N-Terminal Kinase-3
**GSK3β**	Glycogen Synthase Kinase-3 Beta
**QED**	Quantitative Estimate of Drug-Likeness
**SA**	Synthetic Accessibility
**VAE**	Variational Autoencoder
**RNN**	Recurrent Neural Network
**GNN**	Graph Neural Network
**MOSES**	Molecular Sets (Benchmark Platform)
**SEA**	Similarity Ensemble Approach
**MaxTC**	Maximum Tanimoto Coefficient
**ECFP4**	Extended Connectivity Fingerprint (Radius 4)
**ADMET**	Absorption, Distribution, Metabolism, Excretion, and Toxicity
**BRICS**	Breaking of Retrosynthetically Interesting Chemical Substructures
**SNN**	Nearest Neighbor Similarity
**IntDiv**	Internal Diversity
**Frag**	Fragment Similarity
**FDA**	Food and Drug Administration
**DL**	Distillation Learning (Baseline Model)
**DM**	Distillation Model (Baseline Model)
**MCMGL**	Multi-Constraint Molecular Generation (Light Version)
**MCMGM**	Multi-Constraint Molecular Generation (Medium Version)
**Semi-MCMGM**	Semi-Supervised Multi-Constraint Molecular Generation (Medium Version)
**TY3H**	Tyrosine Hydroxylase
**ADCY5**	Adenylate Cyclase 5
**NSFC**	National Natural Science Foundation of China
