# Supplementary material for: Multi-Property De Novo Drug Design Using Deep Learning-Based Knowledge Distillation and Reinforcement Learning
Source: Int J Mol Sci. 2026 Jul 8;27(14):6125. doi: 10.3390/ijms27146125 (PMC13410276; doi:10.3390/ijms27146125)
Supplement: Supplementary file 1 [file ijms-27-06125-s001.zip › ijms-4392765-supplementary.pdf]

Supplementary Materials

Results for JNK3/GSK3β-targeting molecule generation task

The advantages of teacher–student model interaction

Table S1 | The MOSES metrics of the 5000 molecules generated by init-T, init-S, and init-TS (different versions of TSItransRL).

Model	Validity↑	SNN↓	Frag↑	Novelty↑	IntDiv↑	Success↑
init-T	0.808	0.352	**0.972**	0.999	**0.873**	8.9%
init-S	**1.000**	**0.273**	0.881	**1.000**	**0.848**	0.0%
init-TS	**0.994**	0.498	0.807	**0.998**	0.696	**83.4%**

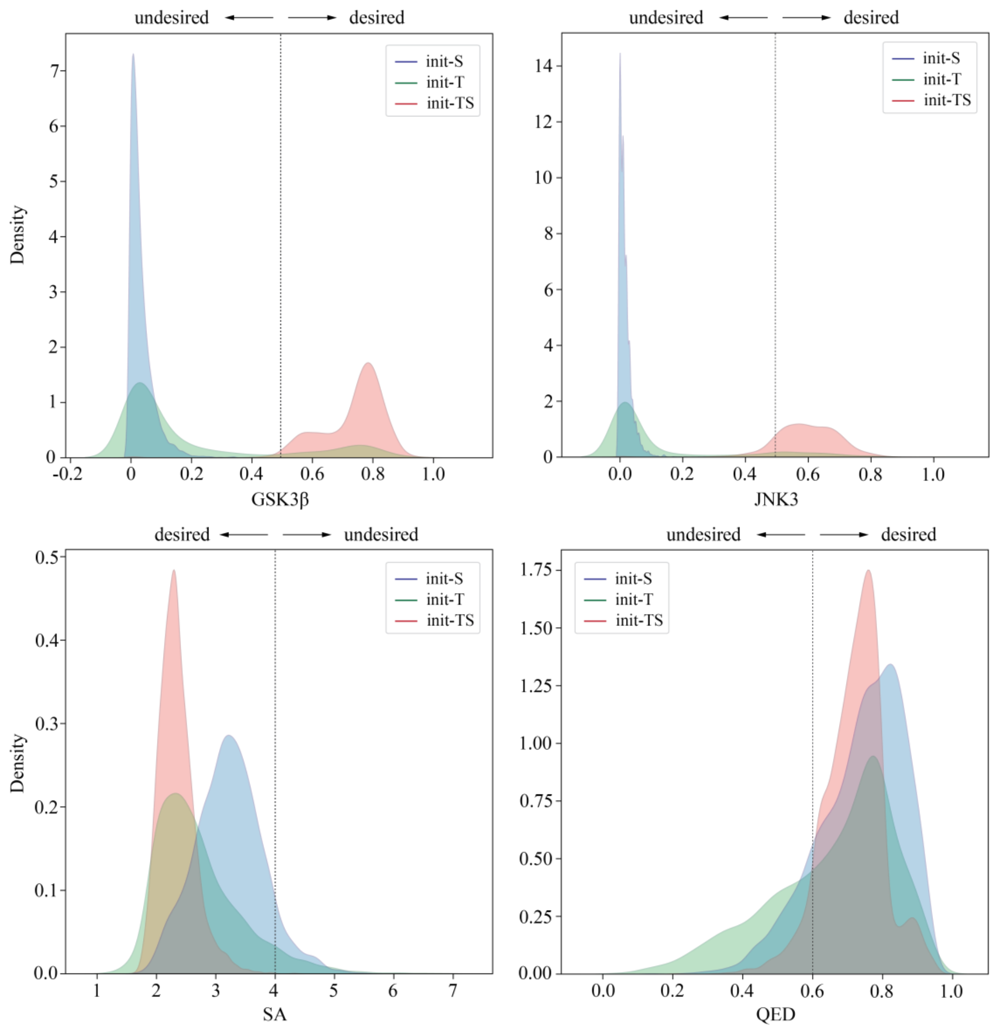

Figure S1 | The advantage of teacher–student interactive knowledge distillation. Distributions of the molecular properties for the molecules generated by init-S, init-T and init-TS.

The teacher–student model interaction analysis.

Table S2 | The MOSES metrics of the 5000 molecules generated by the teacher model (T-model) and student model (S-model) during the knowledge interaction process.

Model	Round	Validity↑	SNN↓	Novelty↑	IntDiv↑	Success↑
	1	0.805±0.003	0.354±0.002	0.999±0.001	0.872±0.001	0.084±0.003
	2	0.969±0.001	0.463±0.001	1.000±0.000	0.709±0.001	0.809±0.010
	3	0.987±0.0004	0.447±0.001	1.000±0.000	0.697±0.001	0.904±0.006
T-model	4	0.989±0.001	0.437±0.001	1.000±0.000	0.694±0.001	0.913±0.003
	5	0.991±0.001	0.432±0.001	1.000±0.000	0.690±0.001	0.924±0.003
	6	0.992±0.001	0.425±0.001	1.000±0.000	0.689±0.001	0.939±0.002
	7	0.994±0.002	0.420±0.001	1.000±0.000	0.686±0.000	0.938±0.003
	1	1.000±0.000	0.273±0.001	1.000±0.000	0.847±0.001	0.000±0.000
	2	0.995±0.0002	0.500±0.001	0.998±0.001	0.697±0.001	0.837±0.008
	3	0.999±0.0003	0.453±0.001	1.000±0.000	0.695±0.0002	0.914±0.005
S-model	4	0.999±0.0004	0.443±0.001	1.000±0.000	0.685±0.001	0.945±0.003
	5	0.999±0.0004	0.432±0.001	1.000±0.000	0.687±0.002	0.938±0.003
	6	1.000±0.0003	0.424±0.002	1.000±0.000	0.688±0.001	0.944±0.002
	7	1.000±0.0001	0.414±0.001	1.000±0.000	0.684±0.001	0.947±0.004

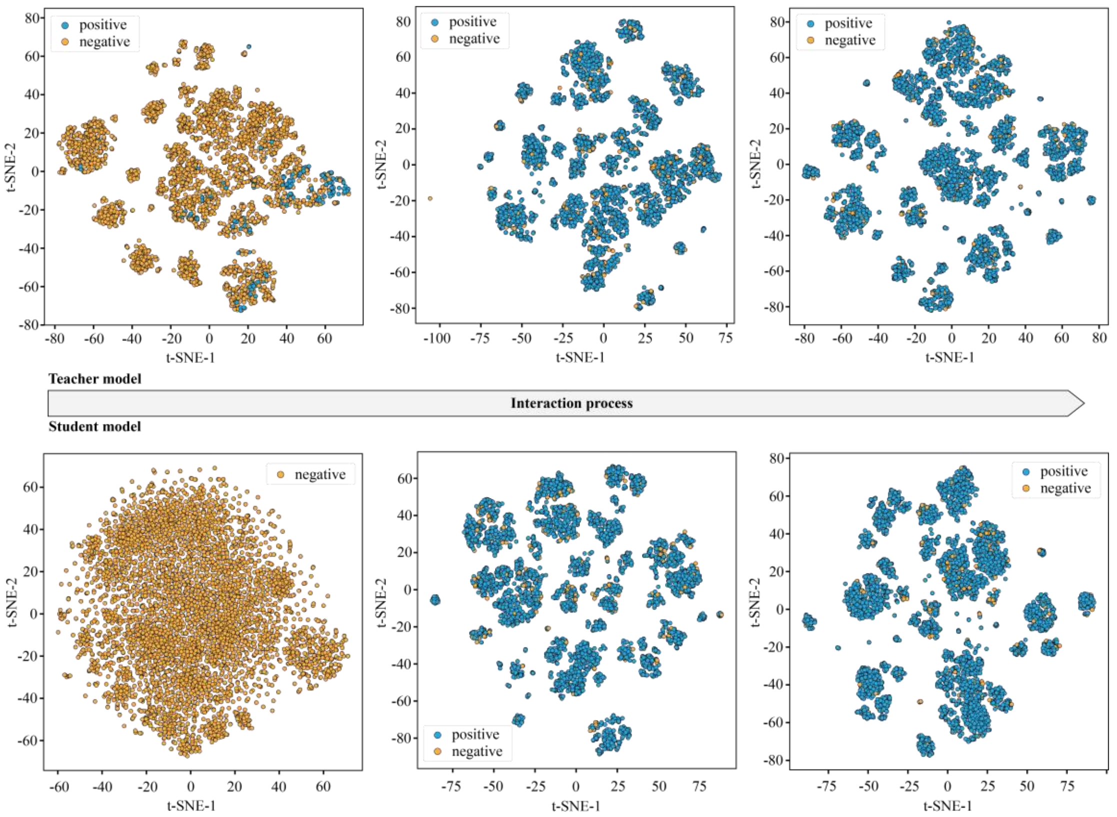

Figure S2 | Visualization of molecular representation space during knowledge interaction. a-c, d-f: The molecular representation of the molecules generated by the teacher model and student model, respectively, at the initial, 4th and 7th rounds of interaction.

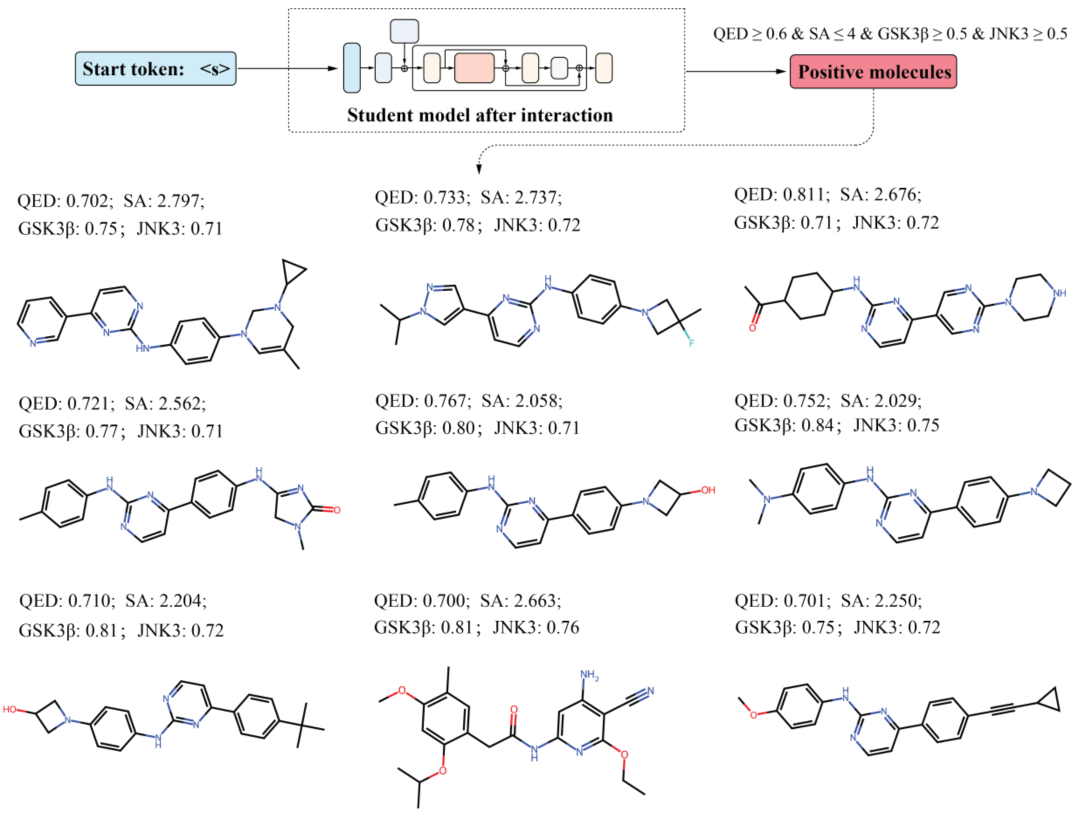

Figure S3 | Novel positive molecules randomly generated by the distilled student model. For the JNK3/GSK3β-targeting molecule generation task, nine novel molecules satisfying JNK3, GSK3β, QED and SA properties were randomly selected and displayed.

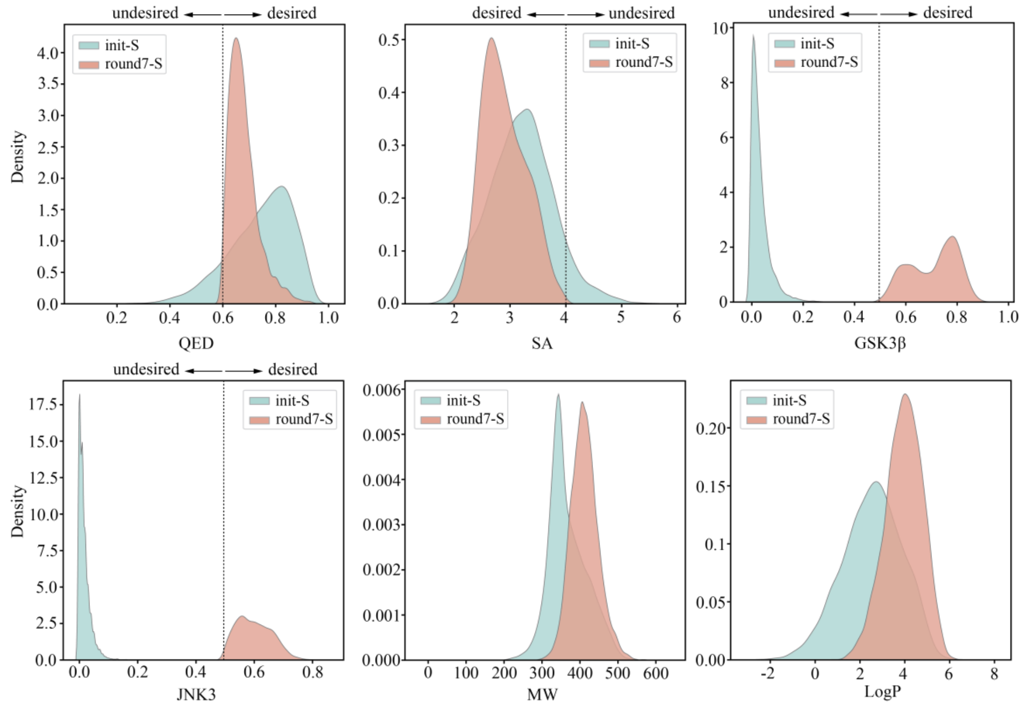

Figure S4 | The distributions of the molecular properties for the molecules generated by

init-S and round7-S. init-S and round7-S are the student model after the first and seventh rounds of interactive knowledge distillation, respectively.

Student model readjustment by RL system.

Table S3 | The conditional and MOSES evaluation metrics of the successful molecules generated by TSItransRL and prior arts.

	Models	Reinvent	Reinvent2.0	MCMGL	MCMGM	Semi-MCMGM	TSItransRL
Conditional metrics	Novelty↑	64.50%	26.80%	36.20%	71.40%	70.30%	**76.82%**
	Div↑	0.671	0.620	0.630	0.686	0.679	**0.718**
MOSES metrics	Unique↑	0.841	0.533	0.085	0.852	0.856	**1.000**
	Frag↑	0.552	0.507	0.514	0.577	0.551	**0.710**
	SNN↓	**0.376**	0.393	0.397	0.382	**0.376**	0.433
	IntDiv↑	0.612	0.563	0.343	0.626	0.619	**0.657**
	Novelty↑	**1.000**	**1.000**	0.999	**1.000**	**1.000**	**1.000**

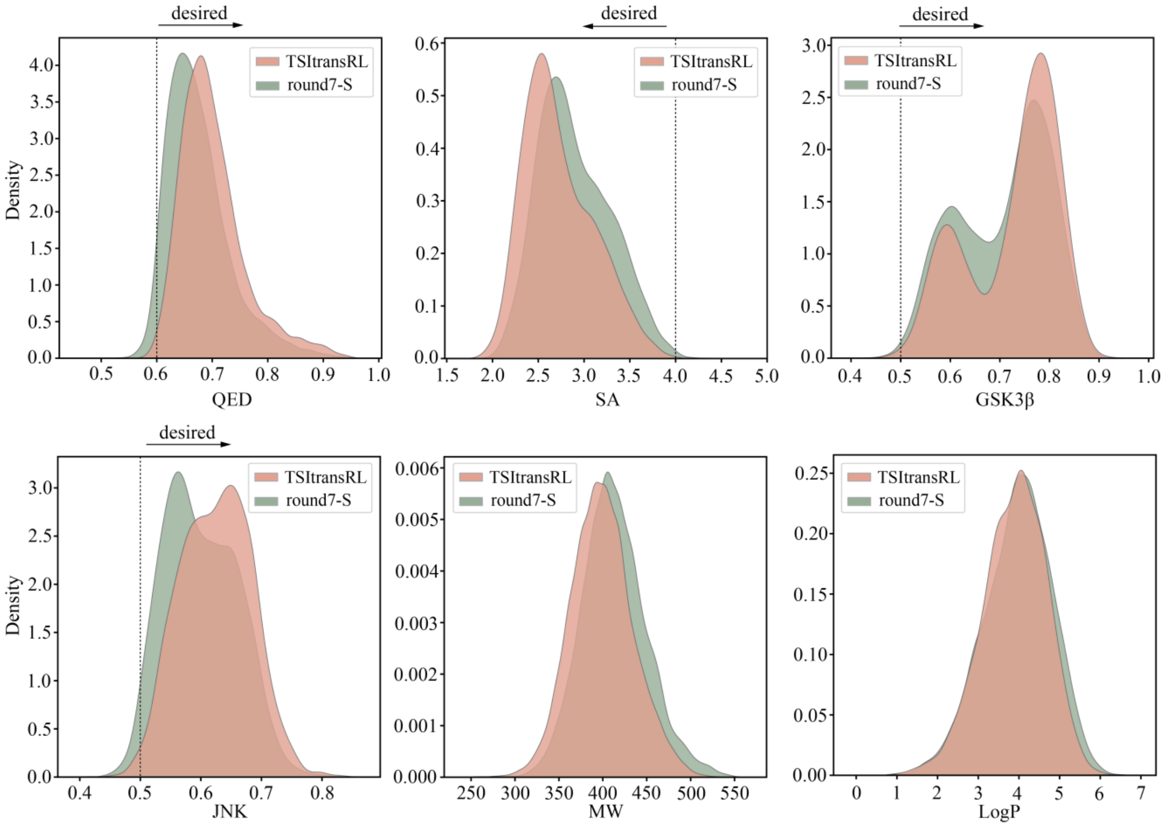

Figure S5 | The distributions of the molecular properties for the molecules generated by round7-S and TSItransRL. TSItransRL is the student model after seven rounds of interactive knowledge distillation and further fine-tuned by reinforcement learning.

Docking analysis of molecules generated by TSItransRL.

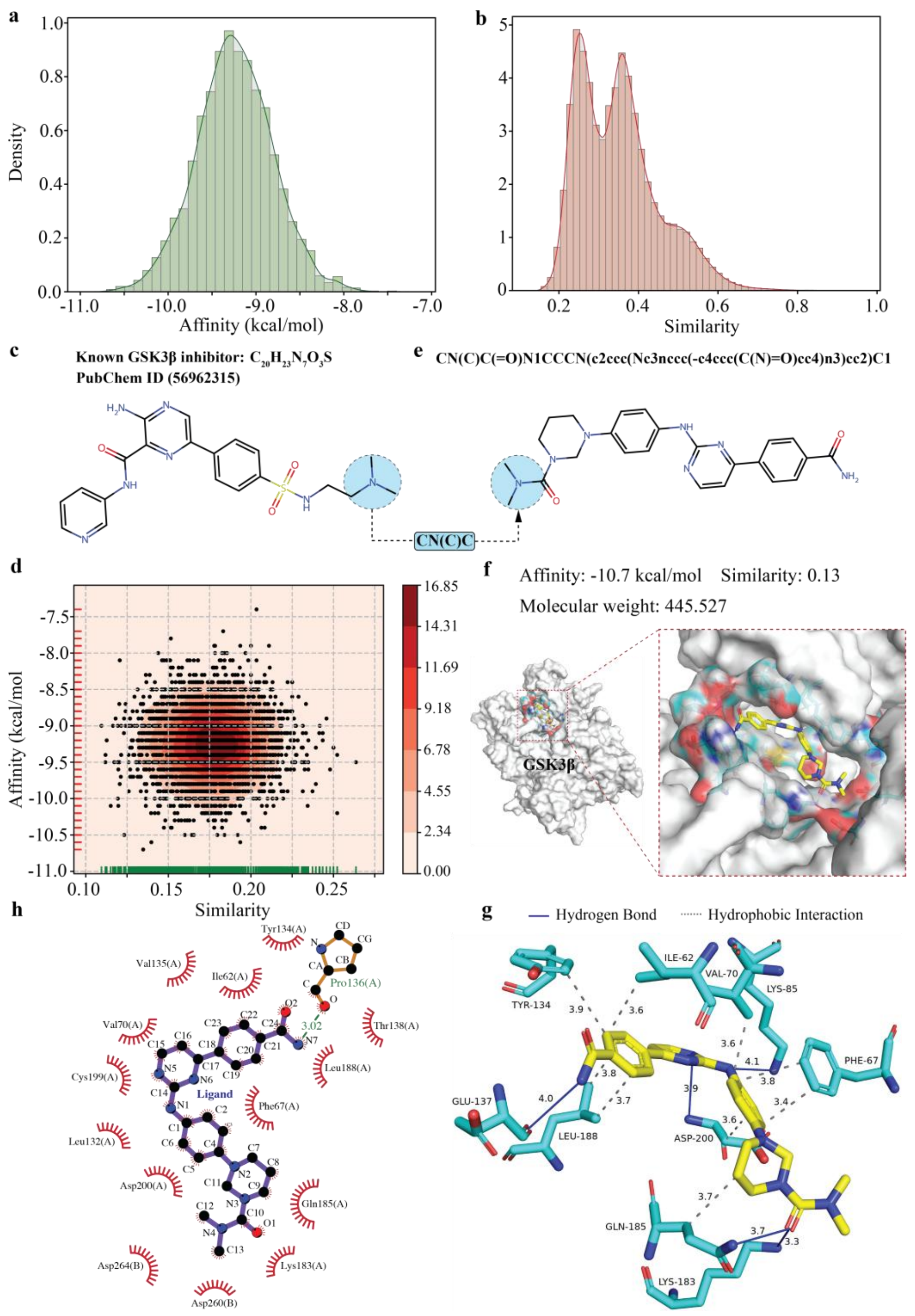

Figure S6 | Docking analysis for the generated molecule and GSK3β. a The distribution of the binding energy with GSK3β for the 5000 molecules generated by TSItransRL using SMILES fragments of known GSK3β inhibitor $C_{20}H_{23}N_7O_3S$（PubChem ID：56962315）as the template. **b** The distribution of the internal Tanimoto similarity for these molecules. **c** Generating novel GSK3β-targeting molecules with more desirable properties from the template. **d** The relationship between the binding energy and the external similarity of these molecules with $C_{20}H_{23}N_7O_3S$. **e** The generated molecule with the lowest affinity score. **f-h** The docking results of the generated molecule with the lowest binding energy.

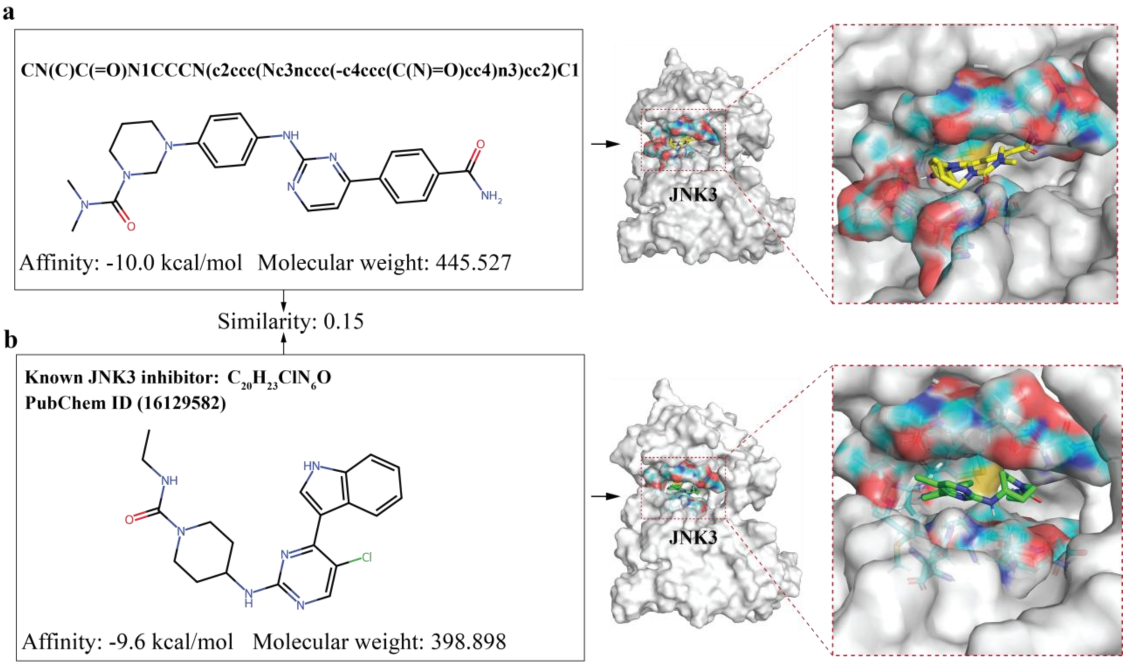

Figure S7 | Docking analysis for molecules and JNK3. a The docking analysis for the generated molecule above and JNK3. **b** The docking analysis for the known JNK3 inhibitor $C_{20}H_{23}ClN_6O$ (PubChem ID: 16129582) and JNK3.

Similarity ensemble approach (SEA) analysis.

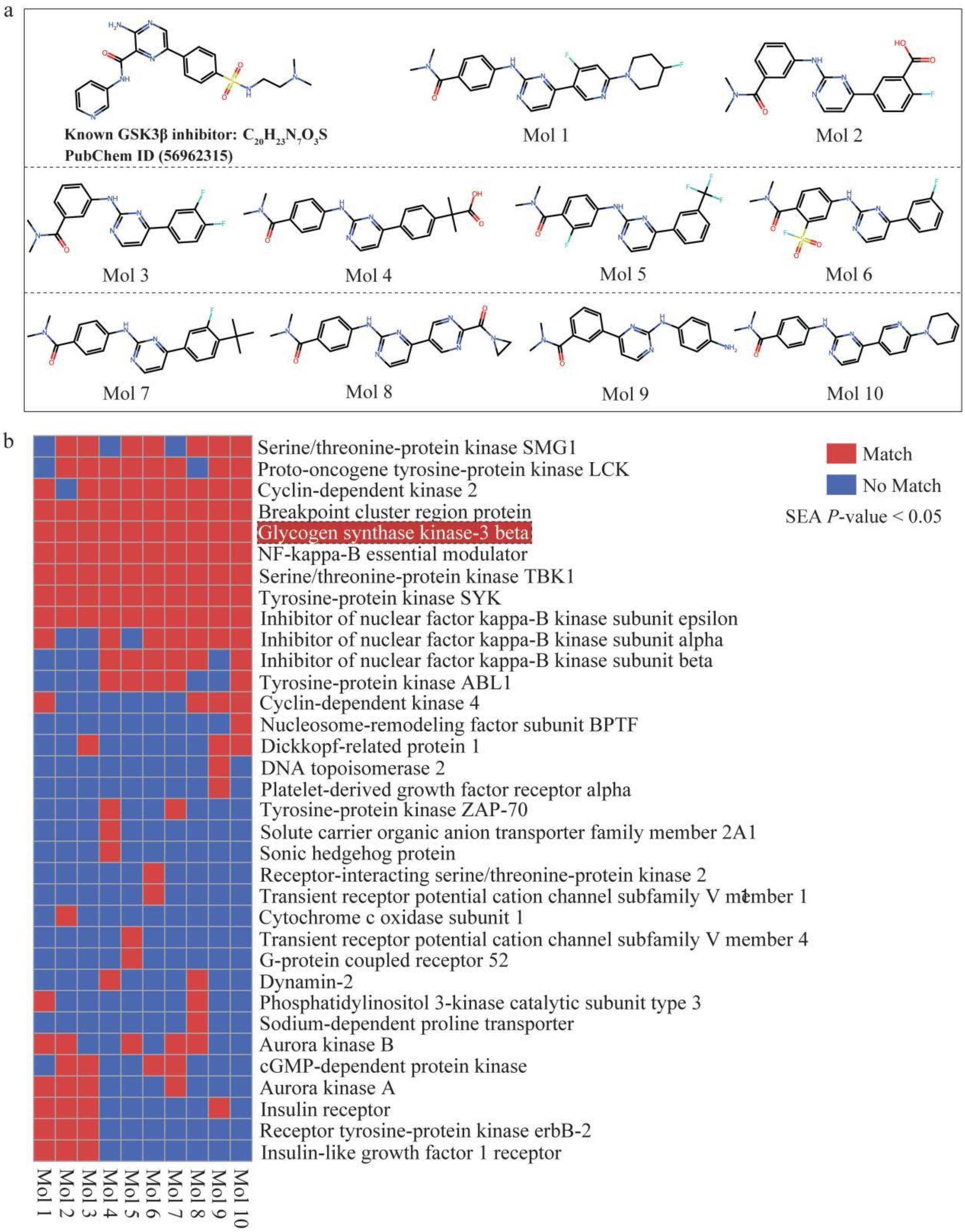

Figure S8 | The SEA analysis for the molecules generated by TSItransRL. a Ten molecules randomly sampled from the novel molecules generated by TSItransRL. **b** The heatmap of the molecules and the potential targets.

Scaffold analysis of the generated molecules.

Table S4 | The similarity between the scaffolds of real active molecules and generated molecules.

Models	Scaffold similarity			
	≤0.1	≤0.2	≤0.3	≤0.4
**REINVENT**	0	0	5	124
**REINVENT2.0**	0	0	1	61
**MCMGL**	0	0	3	17
**MCMGM**	0	0	20	213
**semi-MCMGM**	0	0	22	299
**TSItransRL**	0	0	**139**	**2186**

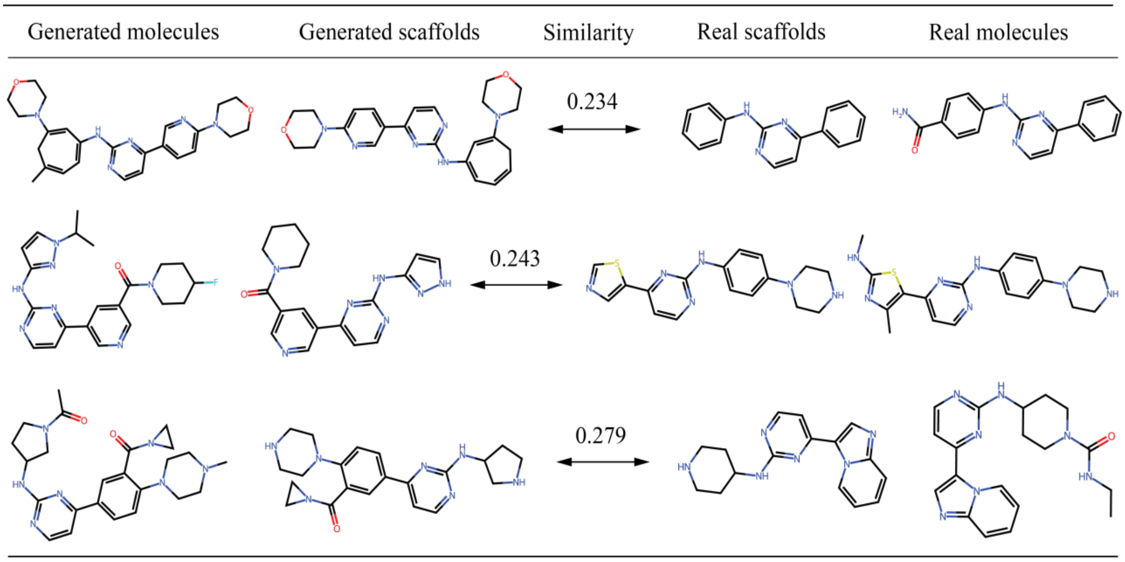

Figure S9 | The top differential scaffolds of the JNK3/GSK3β-targeting molecules generated by TSItransRL. The first column shows the molecules generated by TSItransRL, the second column shows the scaffolds of these molecules, the third column shows the similarity between the scaffolds of the generated molecules and the real molecules, and the fourth and fifth columns show the scaffolds of the molecules from a real activity dataset and the real molecules, respectively.
